# A Chemical Strategy for the Preparation of Multimodified Peptide Imaging Probes

**DOI:** 10.1021/acs.joc.3c00014

**Published:** 2023-03-29

**Authors:** Lucia De Rosa, Ivan Hawala, Rossella Di Stasi, Rachele Stefania, Martina Capozza, Donatella Nava, Luca Domenico D’Andrea

**Affiliations:** † Istituto di Biostrutture e Bioimmagini, Consiglio Nazionale Delle Ricerche, Via Pietro Castellino 111, 80131 Napoli, Italy; ‡ Centro di Imaging Molecolare, Dipartimento di Biotecnologie Molecolari e Scienze per La Salute, Università di Torino, via Nizza 52, 10126 Torino, Italy; § Dipartimento di Scienze Farmaceutiche, Università di Milano, Via Venezian 21, 20133 Milano, Italy; ⊥ Istituto di Scienze e Tecnologie Chimiche “G. Natta”, Consiglio Nazionale Delle Ricerche, Via M. Bianco 9, 20131 Milano, Italy

## Abstract

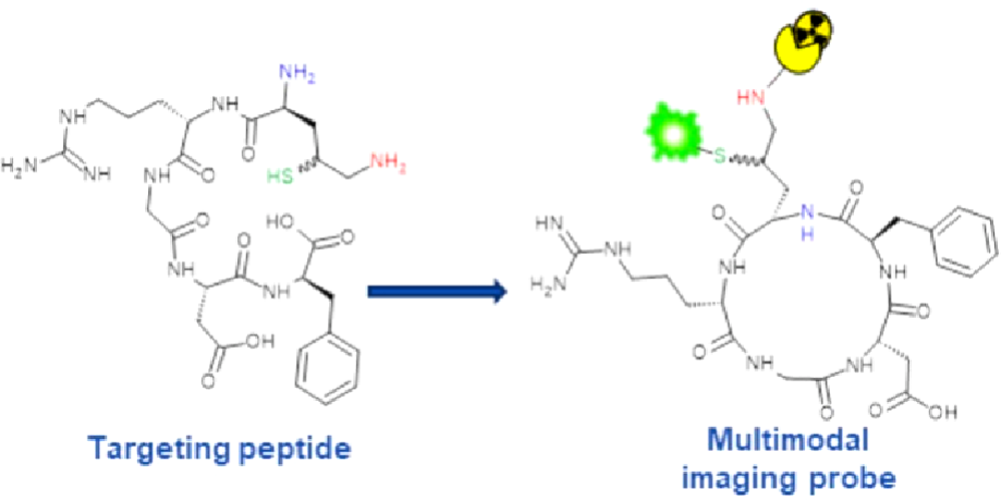

Multimodality probes appear of great interest for innovative imaging applications in disease diagnosis. Herein, we present a chemical strategy enabling site-specific double-modification and cyclization of a peptide probe exploiting native chemical ligation (NCL) and thiol-maleimide addition. The synthetic strategy is straightforward and of general applicability for the development of double-labeled peptide multimodality probes.

## Introduction

Peptides are unquestionably recognized as valuable tools in biomedicine, showing countless applications as new generation drugs endowed with high effectiveness, high selectivity, and low immunogenicity.^1−3^ Tens of bioactive peptides reached the clinic in recent years, and many others are under clinical evaluation as novel therapeutic molecules.^4^ The application of peptides in the diagnostic field is attracting increasing attention as well, especially in oncology.^5,6^ In this latter context, peptides able to selectively target tumor antigens and conjugated to imaging molecular probes, such as fluorophores or radionuclides, can be exploited as homing units for the targeted delivery of the imaging agents to the tumor site. For example, the somatostatin receptor targeting peptide octreotide labeled with radionuclides, such as ^111^In, ^68^Ga, and ^177^Lu, was approved for cancer imaging and radiotherapy, while other peptide-based probes are proceeding in clinical trials such as ^18^F-labeled cyclic (Arg–Gly–Asp) (*cyclo*RGD), which is under investigation for application in cancer and carotid stenosis imaging.^7−10^ The collection of peptide-based imaging probes is expected to rapidly spread as they feature remarkable advantages as diagnostic tools with respect to the use of the antibody-based molecules for targeted imaging. Small size peptides ensure higher tissue/tumor penetration and a faster excretion through renal clearance, overcoming the major limitations associated with the use of antibody-based probes while retaining the benefits in terms of sensitivity and signal-to-noise ratio associated with the targeted distribution of the imaging probe.^5^ Noteworthy, the preparation of peptide-based bioimaging probes can take advantage of the rich chemical toolbox of peptide chemistry that enables their straightforward synthesis and functionalization, strongly expanding their potential applications and utility. Peptide functionalization is usually performed in solid phase during on-resin peptide synthesis, and it often requires complex orthogonal protection/deprotection schemes.^11−13^ Peptide functionalization in solid phase, although ensuring a high level of specificity, usually proceeds under harsh conditions, and it is not always well tolerated by the imaging moieties; furthermore, it is expensive due to the consumption of large excesses of precious reactants and it is not environmentally sustainable, being that the synthetic steps are completely performed in organic solvents. The development of cost-effective and greener chemical procedures enabling the site-specific functionalization of peptide sequences under mild conditions is strongly desired to further the effective wide spreading of peptide-probes in bioimaging. In this context, we recently reported an innovative chemical approach for the site-specific dual functionalization, carried out in aqueous buffer at neutral pH, of peptides targeting tumor antigens with both an optical and a PET tag.^14^ Dual imaging probes appear of great interest in the diagnostic field for frontier applications in multimodality imaging, a modern diagnostic methodology that crosses imaging data obtained through different techniques and furnishes complementary information, thus providing the right solution to overcome the limitations of each imaging technique. In this paper, we expand the potential and the suitability of the previously described peptide labeling approach by presenting a chemical strategy that enables cyclization and double-modification of a peptide probe completely performed in solution. This stepwise strategy consists of two native chemical ligation (NCL) reactions and a thiol-maleimide addition.^15,16^ As proof-of-concept, we applied the strategy to the well-established tumor targeting cyclic peptide *cyclo*RGD.^17^ In this novel approach, the first NCL was exploited to pursue head-to-tail peptide cyclization, the second NCL step allowed the conjugation of the AAZTA (6-amino-6-methylperhydro-1,4-diazepinetetraacetic acid)^18^ metal chelator, and, finally, the third functionalization step was performed through Michael addition using a cyanine 5.5 maleimide fluorophore. AAZTA is a polyaminopolycarboxylateheptadentate chelator that can complex ^68^Ga as PET radionuclide, while cyanine 5.5 is a fluorescent reporter useful for optical imaging (OI).^19−22^ All functionalization steps were performed on the resin-cleaved peptide, conveniently proceeded in aqueous solution, which required mild conditions using a limited excess of the reactive probes. Notably, the reported chemical approach may be readapted for the introduction of three molecular handles on a linear peptide, further expanding its potential utility. The functionalization strategy is straightforward, economically and environmentally more sustainable than classical approaches, and it appears of general applicability for the development of peptide probes with applications in multimodality imaging. Conveniently, the three chemical modifications are gathered on peptide N-terminal position, leaving the targeting motifs exposed and available to establish molecular interactions.

## Results and Discussion

**Figure 1:**
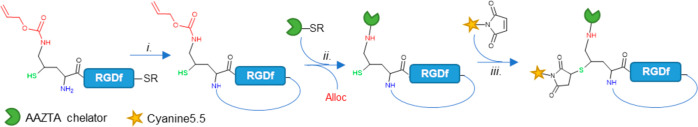
Scheme of the chemical strategy designed to obtain a cyclic, double labeled imaging peptide probe: *i*. first modification, intramolecular cyclization by NCL; *ii*. second modification, labeling with AAZTA by NCL; *iii*. third modification, labeling with cyanine 5.5 by thiol-maleimide addition.

**Figure 2:**
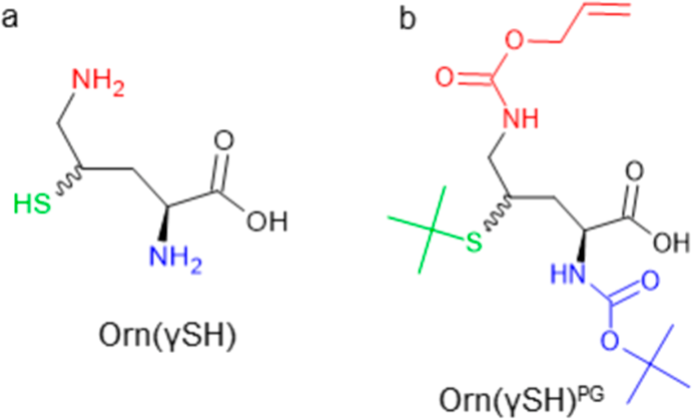
(a) Chemical structure of the unnatural amino acid γ-(*R*,*S*)-mercapto-l-ornithine, Orn(γSH), unprotected and (b) with protecting groups, Orn(γSH)^PG^.

The chemical strategy conceived to afford peptide functionalization in solution is schematically depicted in Figure 1. The α_v_β_3_ integrin receptor targeting cyclic peptide based on the Arg-Gly-Asp sequence (*cyclo*RGD) was used as the model molecule due to its proven utility in the targeting of tumor vasculature for diagnostic applications.^10,23^ In the previous strategy reported by us, the Arg–Gly–Asp–d–Phe–Lys peptide was synthesized and head-to-tail cyclized in solid phase.^14^ After cyclization, the side-chain of the Lys was selectively deprotected in solid phase and a Cys residue was coupled on the ε-amino group of the Lys. Subsequently, the peptide was deprotected and cleaved from the resin, and the Cys residue was exploited to conjugate in solution the AAZTA chelator and the cyanine 5.5 fluorophore, respectively, via NCL and thiol-maleimide chemistry, using a thioester derivative of the metal chelator and a maleimide-conjugated dye. The improved strategy described in this work takes advantage of the use of an unnatural amino acid, a γ-mercapto-ornithine (Orn(γSH) (Figure 2a), as Cys surrogate, to replace Lys residue overall reducing the length of the linker.

The peptide was synthesized in solid-phase as C-terminal thioester, and the Orn(γSH) amino acid, suitably protected, was coupled at the N-terminal position. The nonproteinogenic amino acid enables the straightforward cyclization and double labeling of the target peptide after a selective deprotection and performing two consecutive NCL reactions and a thiol-maleimide addition. The first NCL reaction, performed on the peptide after cleavage from the resin and involving the C-terminal thioester, the γ-thiol and α-amine of Orn(γSH), was exploited to afford the first reaction in solution, i.e., peptide head-to-tail cyclization. Once the peptide was cyclized, the δ-amine function of Orn(γSH) was deprotected in solution, becoming available for the second reaction, driven by NCL reaction. In this second NCL step, the γ-thiol and the δ-amine group of Orn(γSH) reacted with a thioester derivative of the AAZTA chelator. Finally, the third site-specific reaction was a thiol-maleimide addition, exploiting the γ-thiol of Orn(γSH) to conjugate the maleimide derivative of the cyanine 5.5 fluorophore to the targeting peptide.

Protecting groups (PGs) for the thiol- and amino-functions of Orn(γSH) were chosen considering that the γ-thiol of Orn(γSH) should be conveniently deprotected during peptide cleavage from the resin, as well as the α-amine, while the δ-amino group of Orn(γSH) requires an orthogonal protection selectively removable in solution on the cyclic peptide. Accordingly, we selected the *tert*-butyloxycarbonyl (Boc) for the α-amine, the allyloxycarbonyl (Alloc) for δ-amine, and *tert*-butyl (*t*Bu) for the γ-thiol (*N*^α^-Boc-Orn(*N*^δ^-alloc, γ-mercapto-*t*Bu)–OH, Orn(γSH)^PG^, Figure 2b). Boc and *t*Bu are acid-sensitive groups that are removed during the cleavage from the resin in trifluoromethanesulfonic acid (TFMSA)/trifluoroacetic acid (TFA).^24,25^ Alloc group is stable to acid treatment, and it is selectively removed by palladium-catalyzed reductive deprotection.^26,27^ Therefore, we set the synthetic procedure for the preparation of Orn(γSH)^PG^ in analogy to that reported for δ-(*R*,*S*)-mercaptolysine by Kumar et al.^28^ The synthesis (Scheme S1) consisted of 11 reactive steps, starting from l-aspartic acid.

**Figure 3:**
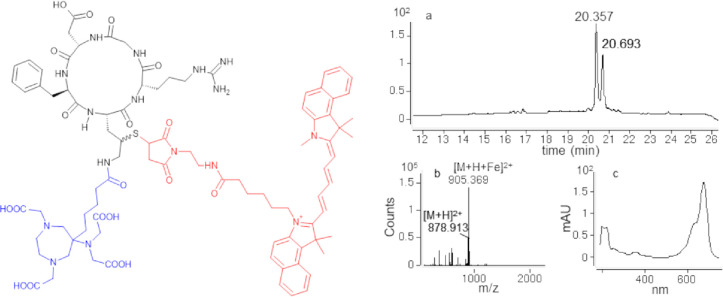
Left panel: chemical structure of the multimodal probe *cyclo*(-Orn(*N*^δ^-CO-C4-AAZTA,γ-*S*-succinimido-Cy5.5)-Arg-Gly-Asp-d-Phe-). *Cyclo*RGD targeting peptide is reported in black, the AAZTA-C4-CO chelator is represented in blue, the cyanine 5.5-maleimide moiety is highlighted in red. Right panel: LC–MS analysis of *cyclo*(-Orn(*N*^δ^-CO-C4-AAZTA, γ-*S*-succinimido-Cy5.5)-Arg-Gly-Asp-d-Phe-). (a) Chromatographic profile revealed at 210 nm; (b) ESI-ToF mass spectrum of the two peaks eluted at ∼20 min. MW^th^: 1755.717 Da; MW^exp^ monoisotopic: 1755.809 and 1808.720 Da (+53 Da) ascribable to the chelation of a Fe(III) ion by AAZTA during the LC–MS analysis. Cyanine 5.5 harbors a quaternary nitrogen that confers an extra +1 net charge to the parental ion.

Orn(γSH)^PG^ was obtained with a final overall yield of 4% as a racemic mixture of the *R* and *S* isomers at the C^γ^ chiral center, according to previously reported data (Figure S1–S10).^28^ A C-terminal thioester derivative of the targeting peptide was prepared by Fmoc chemistry, exploiting the *N*-(2-hydroxy-5-nitrobenzyl) cysteine (*N*-Hnb-Cys) as thioesterification device.^29−31^ The amino acid Orn(γSH)^PG^ was coupled as last amino acid. The C-terminal crypto-thioester derivative of the peptide was deprotected and cleaved from the resin by treatment with a mixture of TFMSA/TFA/thioanisole,^32^ resulting in the effective removal of all protecting groups, except δ-Alloc that is resistant to acid treatment. The peptide was intramolecularly cyclized by NCL by dissolving the crude product in 0.2 M phosphate buffer pH 7.1, 3.0 M guanidinium hydrochloride, 25 mM tris-carboxyethylphosphine (TCEP), 5.0 M imidazole. Imidazole was added to the mixture as effective NCL catalyst.^33^ LC–MS analysis of the NCL mixture after 16 h showed that the peptide completely reacted, yielding the head-to-tail cyclized molecule. Intramolecular NCL was effectively favored over peptide multimerization, as only trace amounts of the peptide multimers were revealed in the LC–mass trace. Notably, α-amine and γ-thiol group of Orn(γSH) reacted efficiently in the NCL reaction, according to literature data suggesting that a 1,3-amino thiol compound shows a reaction rate in NCL comparable to that of a 1,2 amino-thiol compound, such as a N-terminal Cys.^34^ NCL mixture was purified by RP-HPLC, affording pure *cyclo*(-Orn(*N*^δ^-alloc, γ-mercapto)-Arg-Gly-Asp-d-Phe-) peptide (MW^th^: 705. 470 Da; MW^exp^: 705.293 Da; Figure S11). Then the Alloc group was selectively removed to expose the free δ amino group, obtaining the fully unprotected *cyclo*(-Orn(γ-mercapto)-Arg-Gly-Asp-d-Phe-) peptide. The purified peptide was eluted as double peak (Figure S12a) both showing the mass value of *cyclo*(-Orn(γ-mercapto)-Arg-Gly-Asp-d-Phe-) (MW^th^ monomer: 621.389 Da; MW^exp^ monomer: 621.271 Da), according with the presence of two diastereomers. The peptide appeared in part as a disulfide bonded dimer. The treatment of the sample with the reducing agent TCEP allowed conversion of the dimer species into the monomer (Figure S12b). *cyclo*(-Orn(γ-mercapto)-Arg-Gly-Asp-d-Phe-) peptide was directly used as a mixture of dimer and monomer in the second modification step, as the TCEP present in the NCL buffer ensured the reduction of the thiol group of Orn(γSH) and its full availability for the reaction. A 2-mercaptoethanesulfonate (MES) thioester derivative of the metal chelator AAZTA-C4-COOH (AAZTA-C4-CO-MES)^14^ was conjugated to *cyclo*(-Orn(γ-mercapto)-Arg-Gly-Asp-d-Phe-) by NCL. A slight excess (1.5 equiv) of AAZTA-C4-CO-MES allowed complete conversion of the peptide in the AAZTA-conjugate *cyclo*(-Orn(*N*^δ^-CO-C4-AAZTA, γ-mercapto)-Arg-Gly-Asp-d-Phe-) (MW^th^: 1049.811 Da; MW^exp^: 1103.351 Da; +53 Da, ascribable to the chelation of a Fe(III) ion by AAZTA during the LC–MS analysis) (Figure S13). The γ-thiol of Orn, after NCL reaction, is in the free form and it was exploited for the third modification step. A thiol-maleimide addition reaction was adopted as convenient thiol selective chemistry because maleimide derivatives of a wide set of fluorophores are commercially available. In particular, *cyclo*(-Orn(*N*^δ^-CO-C4-AAZTA, γ-mercapto)-Arg-Gly-Asp-d-Phe-) was labeled with cyanine 5.5-maleimide. The reaction proceeded quickly in aqueous buffer at pH around neutrality and it was completed within a few hours at room temperature using a slight excess of the fluorophore (1.5 equiv). The final, double labeled imaging peptide probe *cyclo*(-Orn(*N*^δ^-CO-C4-AAZTA, γ-*S*-succinimido-Cy5.5)-Arg-Gly-Asp-d-Phe-) was purified by RP-HPLC and obtained with high purity (Figure 3). The AAZTA chelator can be complexed with gallium under mild conditions by treatment with 1 equiv of GaCl_3_ in acidic aqueous buffer, at room temperature.^14,20^*cyclo*(-Orn(*N*^δ^-CO-C4-AAZTA, γ-*S*-succinimido-Cy5.5)-Arg-Gly-Asp-d-Phe-) was successfully complexed with Ga(III) ion by treatment with an equimolar amount of GaCl_3_ in acetate buffer pH 4.6/CH_3_CN (1:1), at room temperature for 1 h (Figure S14) to perform cell-binding studies using a probe with the same chemical properties of the PET/OI imaging probe.

**Figure 4:**
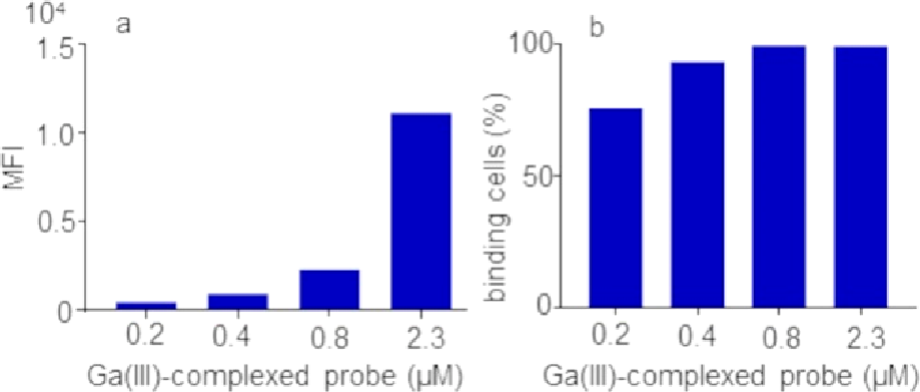
Binding analysis by flow cytometry of Ga-complexed *cyclo*(-Orn(*N*^δ^-CO-C4-AAZTA, γ-*S*-succinimido-Cy5.5)-Arg-Gly-Asp-d-Phe-) probe to U-87 MG cells overexpressing αvβ3 integrin receptor. Histogram plots of (a) mean fluorescence intensity (MFI) and (b) percentage of cells revealed by FACS analysis of U-87 MG cells after incubation for 1 h on ice with the probe 0.2, 0.4, 0.8, and 2.3 μM.

Metal complexation procedure did not alter fluorophore spectroscopic properties, as the final Ga(III)-complexed probe featured the UV–vis spectrum of the parent cyanine 5.5 maleimide (Figure S15). Flow cytometry experiments performed using human glioblastoma U-87 MG cells overexpressing αvβ3 integrin receptor confirmed the ability of the final peptide probe to effectively target αvβ3 expressing tumor cells in a dose-dependent manner (Figure 4).

## Conclusions

Herein a straightforward procedure to prepare multiple modified peptide probe for multimodal imaging is reported. Advantageously, the chemical modifications are performed in solution, using chemo-selective reactions under mild conditions. As proof of concept, the strategy was applied to the preparation of a cyclic tumor targeting peptide labeled with a metal chelator for ^68^Ga radiolabeling and a fluorophore, providing a good candidate probe for PET/OI dual modality imaging.

## Experimental Section

### Synthesis of *N*^α^-Boc-Orn(*N*^δ^-alloc,γ-mercapto-tBu)–OH

#### Materials and Methods

All reagents were purchased by Sigma-Aldrich and IRIS Biotech. All solvents were purchased by VWR International and were used without further purifications. NMR spectra were recorded at 298 K on a Bruker ADVANCE 600 spectrometer. Deuterated chloroform (CDCl_3_) and NMR tube were purchased from Sigma-Aldrich. Mass spectra with electrospray ionization (ESI) were recorded on a SQD 3100 Mass Detector (Waters). The HPLC–MS analytical runs were carried out on a Waters AutoPurification system (3100 Mass Detector 600 Quaternary Pump Gradient Module, 2767 Sample Manager and 2487 UV/Visible Detector). UPLC–MS analyses were performed using a Waters Acquity UPLC H-Class coupled with and ESI source, a quadrupole (QDa) mass analyzer, and dual-wavelength UV/vis TUV Detector.

#### Dimethyl (*S*)-2-*tert*-butoxycarbonylamino-butanodioate (**1**)

In a three-neck round-bottom flask, 5.32 g of l-aspartic acid (39.97 mmol) was dissolved in methanol (CH_3_OH) (120 mL). The solution was brought to 0 °C with an ice bath, and then trimethylsilyl chloride (Me_3_SiCl) (23.2 mL, 4.4 equiv) was added dropwise. The ice bath was removed, and the solution was left to stir overnight at room temperature. 36 mL of triethylamine (TEA) was added to the reaction solution. Subsequently, 9.6 g of di-*tert*-butyl dicarbonate ((Boc)_2_O) (1.1 equiv) was added. The reaction was left to stir overnight at room temperature. The solvent was evaporated *in vacuo*. The obtained white solid was washed with diethyl ether (Et_2_O) (3 × 250 mL) on Celite. The collected supernatant was evaporated, and the crude product was purified with flash silica chromatography with methylene chloride (CH_2_Cl_2_)/CH_3_OH (99:1 to 97:3) to afford pure **1** as white solid (7.23 g, 69%). ^1^H NMR (600 MHz, CDCl_3_) δ 5.52 (d, *J* = 8.2 Hz, 1H), 3.78 (s, 3H), 3.72 (s, 3H), 3.04 (dd, *J* = 17.1, 4.4 Hz, 1H), 2.85 (dd, *J* = 17.1, 4.6 Hz, 1H), 1.47 (s, 9H) (Figure S1).

#### Dimethyl (2*S*)-2-{(*tert*-butoxy)-*N*-[(*tert*-butyl)oxycarbonyl]carbonylamino}butane-1,4-dioate (**2**)

In a three-neck round-bottom flask, 7.23 g of **1** was dissolved in acetonitrile (90 mL). 676 mg of 4-dimethylaminopyridine (DMAP) (0.2 equiv) and 6.64 g of (Boc)_2_O (1.1 equiv) were added to the obtained solution. The reaction was left to stir at room temperature for 2 h. A second portion of (Boc)_2_O was added to the solution (3.02 g, 0.5 equiv) and it was left to stir overnight at room temperature. The solvent was evaporated *in vacuo* and the crude product was purified on flash silica chromatography with petroleum ether/ethyl acetate (8:2) to afford pure **2** as white solid (8.91 g, 89%). ^1^H NMR (600 MHz, CDCl_3_) δ 5.47 (t, *J* = 6.7 Hz, 1H), 3.75 (s, 3H), 3.73 (s, 3H), 3.27 (dd, *J* = 16.4, 7.1 Hz, 1H), 2.76 (dd, *J* = 16.4, 6.4 Hz, 1H), 1.53 (s, 18H) (Figure S2).

#### Methyl (2*S*)-2-{(*tert*-butoxy)-*N*-[(*tert*-butyl)oxycarbonyl]carbonylamino}-4 oxobutanoate (**3**)

In a two-neck round-bottom flask equipped with rubber septum, 4.45 g of **2** was dissolved in dry Et_2_O (125 mL). The solution was brought to −78 °C with acetone/N_2_ bath. 13.56 mL of a 1 M solution of diisobuthylalluminium hydrade (DIBAL) in hexane (12.3 mmol, 1.1 equiv) was added dropwise. After the end of the addition, the solution was left to stir at −78 °C for 5 min and then quenched with H_2_O (1.54 mL, ab. 7 equiv). The solution was stirred for an additional 30 min, and then the solvent was evaporated *in vacuo* and the crude product was purified via flash silica chromatography with petroleum ether/ethyl acetate (95:5 to 85:15) to afford pure **3** (2.58 g, 63%). ^1^H NMR (600 MHz, CDCl_3_) δ 9.82 (s, 1H), 5.55 (t, *J* = 6.2 Hz, 1H), 3.74 (s, 3H), 3.44 (dd, *J* = 17.8, 6.6 Hz, 1H), 2.93–2.77 (m, 1H), 1.53 (s, 18H) (Figure S3).

#### Methyl (5*R*,*S*)-hydroxy-5-nitro-(2*S*)-2-{(tert-butoxy)-*N*-[*tert*-butyloxy carbonyl]-carbonylamino}-pentanoate (**4**)

In a round-bottom flask, 2.58 g of **3** was dissolved in 6.9 mL of nitromethane and the obtained solution was brought to −5 °C with a calcium chloride (CaCl_2_)/ice bath. Under inert atmosphere of argon, 1.23 g of tetra-*n*-butylammonium fluoride (TBAF) (0.5 equiv) was added. The solution was left to stir at 0 °C for 15 min. The solvent was evaporated *in vacuo* and the crude product was purified on flash silica chromatography with petroleum ether/ethyl acetate (8:2 to 7:3) to afford the pure **4** (3.05 g, 73%). ^1^H NMR (600 MHz, CDCl_3_) δ 5.56 (br, 1H), 5.01 (br, 1H), 4.70 (dd, 1H), 4.65 (br, 1H), 3.76 (s, 3H), 2.60 (br, 1H), 2.26 (br, 1H), 2.07 (br, 1H), 1.53 (s, 18H) (Figure S4).

#### Methyl 5-nitro-2-(bis(tert-butoxycarbonyl)amino)-pent-4-enoate (**5**)

In a round-bottom flask, 1.24 g of **4** was dissolved in Et_2_O (15.5 mL), and the solution was brought at 0 °C with an ice bath. 349 μL of acetic anhydride (1.2 equiv) and 148 mg of DMAP (0.4 equiv) were added to the reaction solution. The solution was stirred until the consumption of the starting material, monitored by thin layer chromatography (TLC). The solvent was evaporated *in vacuo* and the crude product was purified on flash silica chromatography with petrol ether/ethyl acetate (85:15) to afford the pure **5** (0.852 g, 72%). The purity of the product was checked by analytical UPLC–MS by employing an ACQUITY UPLC Peptide BEH C18 column (300 Å, 1.7 μm, 2.1 × 100 mm^2^). Eluent: (A) 0.05% TFA in H_2_O, (B) 0.05% TFA in CH_3_CN. Gradient profile; linear gradient from 5% to 50% of B in 7 min, linear gradient from 50% to 100% in 3 min, isocratic at 100% for 3 min. Flow rate of 0.4 mL/min and UV detection at 210 nm. Purity 99%. ESI-MS (*m*/*z*): calcd: For C_16_H_26_N_2_O_8_ (M+Na)^+^ 397.38 found: 397.28. ^1^H NMR (600 MHz, CDCl_3_) δ 7.28 (br, *J* = 9.7 Hz, 1H), 7.06 (br, *J* = 25.7 Hz, 1H), 5.10 (br, 1H), 3.77 (s, 3H), 2.96 (br, *J* = 72.9, 67.9, 33.0 Hz, 2H), 1.52 (s, 18H) (Figure S5).

#### Methyl 5-nitro-2-(bis(tert-butoxycarbonyl)amino)-4-(*tert*-butylthio)pentanoate (**6**)

To a three-neck round-bottom flask under inert atmosphere of argon equipped with a rubber septum, 6.5 mL of dry tetrahydrofuran (THF) was added. 180 μL of *tert*-butyl thiol (*t*-BuSH) (1.602, 1.2 equiv) was added to the solution, which was brought to −10 °C with a sodium chloride (NaCl)/ice bath. Subsequently, 1 mL of a 1.6 M solution of *n*-butyl lithium (*n*BuLi) in hexane (1.602 mmol, 1.2 equiv) was added to the obtained solution, which was left to stir at −10 °C for 10 min. The NaCl/ice bath was removed, and the solution was brought to −78 °C with an acetone/N_2_ bath. 500 mg of **5** (1.335 mmol, 1 equiv), previously dissolved in dry THF, was slowly added to the reaction solution for 10 min. The reaction was left to stir at −78 °C for 1 h and then was quenched with ammonium chloride (NH_4_Cl) (5 mL) and 20 mL of H_2_O was added. The product was extracted with ethyl acetate (3 × 15 mL) and the organic phase was dried with sodium sulfate (Na_2_SO_4_). The solution was filtered, and the solvent was evaporated *in vacuo*. The product has been used for the subsequent step without further purification (**6**, 545 mg, 88%). ESI-MS (*m*/*z*): calcd: For C_20_H_36_N_2_O_8_ (M+H)^+^ 465.57; found: 465.19. ^1^H NMR (600 MHz, CDCl_3_) δ 5.19 (br, *J* = 42.9 Hz, 1H), 4.56 (br, 2H), 4.15 (d, *J* = 6.5 Hz, 1H), 3.75 (s, 3H), 2.45 (br, 2H), 1.53 (s, 18H), 1.37 (s, 9H) (Figure S6).

#### Methyl 5-(((allyloxy)carbonyl)amino)-2-(bis(*tert*-butoxycarbonyl)amino)-4-(*tert*-butylthio)pentanoate (**8**)

In a round-bottom flask, 106.1 mg of **6** (0.228 mmol) was dissolved in 4 mL of CH_3_OH/THF 1:1. 108.6 mg of nickel chloride hexahydrate (NiCl_2_·6 H_2_O) (1.2 equiv) was added, and the solution was left to stir at room temperature for 10 min. 51.75 mg of sodium borohydride (NaBH_4_) (6 equiv) was slowly added portion wise to the solution. The reaction was monitored by TLC until a red spot appeared at the base with addition of ninhydrin. The solvent was evaporated *in vacuo* and the product **7** was used for the subsequent step without further purification.

The unstable obtained primary amine (**7**) was redissolved in THF (4 mL). 95.3 μL of TEA (3 equiv) and 29.1 μL of allyl chloroformate (0.274 mmol, 1.2 equiv) were added to the solution, which was left to stir for 1 h. The solvent was evaporated, and the crude product was purified on gravimetric silica chromatography with petroleum ether/ethyl acetate 8:2 to afford the pure **8** (35 mg, 30%). ESI-MS (*m*/*z*): calcd: For C_24_H_42_N_2_O_8_S (M+H)^+^ 519.67; found: 519.39. ^1^H NMR (600 MHz, CDCl_3_) δ 5.82 (br,1H), 5.26–5.11 (br, 3H), 4.46 (br, 2H), 3.65 (s, 3H), 3.32 (br, 2H), 2.83 (s, 1H), 2.43 (br, 1H), 1.99 (s, 1H), 1.43 (s, 18H), 1.25 (s, 9H) (Figure S7).

#### Methyl 5-(((allyloxy)carbonyl)amino)-2-((tert-butoxycarbonyl)amino)-4-(*tert*-butylthio)pentanoate (**9**)

In a round-bottom flask, 277.7 mg of **8** (0.535 mmol) was dissolved in 10 mL of CH_2_Cl_2_. The solution was brought to 0 °C with an ice bath, and then 5 mL of TFA was added dropwise. After 1 h of stirring at room temperature, 150 mL of saturated NaHCO_3_ was added and the obtained solution was extracted with ethyl acetate (3 × 20 mL). The organic phase was collected, dried with Na_2_SO_4_, filtered, and evaporated *in vacuo*. The obtained oil was redissolved in methanol (5 mL), and 1.44 mL of *N*,*N*-diisopropylethylamine (DIPEA) was added. The solution was brought at 0 °C with an ice bath and 1.1 eq of (Boc)_2_O (348.1 mg, 0.589 mmol). The solution was stirred at 0 °C for 2 h. The solvent was evaporated, and the crude product was purified via flash silica chromatography with hexane/ethyl acetate (85:15 to 8:2) to afford the pure **9** (180 mg, 80%). ESI-MS (*m*/*z*): calcd: For C_19_H_34_N_2_O_6_S (M+H)^+^ 419.55; found: 419.27. ^1^H NMR (600 MHz, CDCl_3_) δ 5.94 (ddt, *J* = 16.2, 10.7, 5.5 Hz, 1H), 5.29–5.21 (m, 2H), 4.72–4.36 (m, 2H), 3.89–3.64 (m, 3H), 3.55–3.37 (m, 1H), 3.34–3.21 (m, 1H), 2.97 (t, *J* = 13.5 Hz, 1H), 1.86 (dd, *J* = 21.1, 5.9 Hz, 1H), 1.62 (d, *J* = 32.3 Hz, 2H), 1.46 (s 9H), 1.36 (s, 9H) (Figure S8).

#### 5-(((Allyloxy)carbonyl)amino)-2-((tert-butoxycarbonyl)amino)-4-(*tert*-butylthio)pentanoic acid (**10**)

Three equiv. of NaOH as powder (51.6 mg) was added to a solution of **9** (180 mg; 0.430 mmol) in H_2_O/isopropanol (*i*-PrOH)1:1 (10 mL). The solution was stirred at room temperature for 6 h. The *i*-PrOH was evaporated *in vacuo* and the pH of the solution was brought to 7 by addition of HCl 1 M. The aqueous residue was extracted with ethyl acetate (3 × 5 mL). The organic phases were collected, dried with Na_2_SO_4_, filtered, and evaporated *in vacuo*. The final product was obtained without further purification as a white solid (**10**, 138.8 mg; 80%). The purity of the product was checked by analytical HPLC–MS. HPLC analysis was performed on an Agilent 1200 Infinity Series (Agilent Technologies) using a LUNA Omega Polar C18 3 μm 300 Å 100 × 2.1 mm^2^ column (Phenomenex), applying a linear gradient of gradient of CH_3_CN (0.1% TFA) in H_2_O (0.1% TFA) from 20% to 70% in 40 min, at a flow rate of 0.2 mL/min, revealing the absorbance at 210 nm. Purity >95%.

For this compound, the 1D and 2D NMR spectra were recorded on a Bruker Advance NEO 400 MHz spectrometer, equipped for a BBI probe, at room temperature in CDCl_3_ with residual solvent peaks as the internal reference. The assignment of chemical shifts was made by using COSY, TOCSY, and HSQC (Figure S9). The spectra were acquired with 2048 points in the F2 direction and 256 points in F1, with 8–16–32 scans, and the mixing time for the TOCSY experiment was 60 ms long. The NMR analysis revealed the presence of two diasteroisomeric forms. The resonances of the minor form, when identified, are reported in italic.

^1^H NMR (CDCl_3_, 400 MHz): δ_H_ 5.92 (m, 1H, −CH=CH_2_), 5.50 (m, 1H, exchangeable with D_2_O, NHC(O)OtBu), 5.43 (m, 1H, exchangeable with D_2_O, NHC(O)OAllyl), 5.33 (br, 1H, −CH=CH_2_), 5.25 (br, 1H, −CH=CH_2_), 4.61 (m, 1H, C^α^H), 4.60 (m, 2H, −OCH_2_CH=CH_2_), 3.47/3.96 (m, 1H, C^δ^H_2_), 3.29/3.24 (m, 1H, C^δ^H_2_), 2.96/3.06 (m, 1H, S–C^γ^H), 1.97/2.11 (m, 1H, C^β^H_2_), 1.97/1.82 (m, 1H, C^β^H_2_), 1.46/1.47 (s, 9H, −OC(CH_3_)_3_), 1.36/1.35 (s, 9H, −SC(CH_3_)_3_) (Figure S10a).

^13^C{^1^H} NMR (CDCl_3_, 100 MHz,): δ_C_ 172.8/174.6 (−COOH), 156.3 (−NHC(O)OAllyl), 153.3 (−NC(O)OC(CH_3_)_3_), 132.6 (−OCH_2_CH=CH_2_), 118.3 (−OCH_2_CH=CH_2_), 80.8 (−OC(CH_3_)_3_), 66.1 (−OCH_2_CH=CH_2_), 51.9 (C^α^), 47.2/45.4 (C^δ^–N), 44.3 (−SC(CH_3_)_3_), 39.7/40.2 (C^γ^-S), 35.3/39.2 (C^β^), 31.4 (−SC(CH_3_)_3_), 28.3 (−OC(CH_3_)_3_) (Figure S10a).

HRMS (ESI/TOF) *m*/*z*: [M + Na]^+^ Calcd for C_18_H_32_N_2_O_6_SNa 427.1981; found 427.1866.(Figure S10b).

### Peptide Synthesis

#### Materials and Methods

Fmoc-Gly–OH, Fmoc-d-Phe–OH, Fmoc-Asp(tBu)–OH, and Fmoc-Arg(Pbf)–OH amino acids were supplied by Iris Biotech. Fmoc-Cys(StBu)–OH was obtained from Merk-Millipore. Rink Amide-ChemMatrix resin was from Biotage. 1-[Bis(dimethylamino)methylene]-1H-1,2,3-triazolo[4,5-*b*]pyridinium 3-oxid hexafluorophosphate (HATU) was purchased from GL Biochem. *N*,*N*-Diisopropylethylamine (DIPEA), acetic anhydride, acetic acid, 2-hydroxy-5-nitrobenzaldehyde, sodium cyanoborohydride, dry dichloromethane (DCM), dry dimethylformamide (DMF), diethyl ether, guanidine hydrochloride, piperidine, trifluoracetic acid (TFA), trifluoromethanesulfonic acid (TFMSA), thioanisole, acetonitrile HPLC grade, tris(2-carboxyethyl)phosphine (TCEP) hydrochloride, phenylsilane, and tetrakis(triphenylphosphine)palladium(0) were obtained from Sigma-Aldrich. Sodium dihydrogen phosphate monohydrate and disodium hydrogen phosphate were from Applichem. DMF for peptide synthesis was purchased from Carlo Erba. Methanol and *N*-methyl-2-pirrolidone (NMP) were from Romil. Imidazole was from Biofroxx. Cyanine 5.5 maleimide was purchased by Lumiprobe GmbH. 2-((5-(6-(Bis(carboxymethyl)amino)-1,4-bis(carboxymethyl)-1,4-diazepan-6-yl)pentanoyl)thio)ethane-1-sulfonate (AAZTA-C4-CO-MES) was prepared as described previously.^14^ Preparative reverse-phase HPLC purifications were performed on a HP 1200 Series (Agilent Technologies) fitted with Phenomenex columns. Mass analyses were run using ultrapurity grade solvents (Romil) on an Agilent 1200 Infinity Series (Agilent Technologies) equipped with a diode array, an electrospray ion source (ESI), and a time-of-flight (ToF) ion mass analyzer, fitted with a column Jupiter Proteo 90 Å 4 μm 50 × 2 mm^2^ (Phenomenex) unless otherwise specified. UV/vis quantification was carried out on a NanoDrop 2000 spectrophotometers (ThermoFisher) using quartz cuvette (Hellma).

#### Solid-Phase Synthesis of the Prothioester Linear Peptide *N*^α^-Boc-Orn(*N*^δ^-alloc,γ-mercapto-tBu)-Arg(Pbf)-Gly-Asp(tBu)-d-Phe-*N-*Hnb-Cys(*StBu*)-Gly-NH_2_

The C-terminal prothioester peptide was prepared in solid-phase, using Fmoc-chemistry, according to previously reported protocols.^29,31^ Peptide synthesis was performed on a Rink Amide-ChemMatrix resin (0.52 mmol/g), on 0.1 mmol scale. All the peptide synthesis reaction steps were performed under shaking, at room temperature. Coupling reactions were carried out using 5 equiv of the Fmoc-protected amino acid, 4.9 equiv of HATU as the coupling agent, 10 equiv of the base DIPEA, for 30 min. After each coupling step, unreacted amine groups were acetylated by treatment with a solution of 0.5 M acetic anhydride, 0.13 M DIPEA in NMP, for 5 min. α-Amine Fmoc protecting group was removed by treating the resin with a solution of 20% piperidine in DMF, 2 × 5 min. Each reaction step was followed by resin washes with DMF (5 × 1 min). After loading of the C-terminal glycine on the resin, the thioesterification device *N*-Hnb-Cys(S*t*Bu) was assembled as previously reported.^29,31^ Fmoc-d-Phe–OH was then coupled on the secondary α-amine group of *N*-Hnb-Cys(StBu) using 2.5 equiv of amino acid, 2.4 equiv of HATU, 5 equiv of DIPEA, twice for 1 h, at room temperature, under stirring. Then the peptide was elongated using the peptide synthesis protocol previously described in this section until the coupling of Fmoc-Arg(Pbf)–OH. At that point, the resin was dried and a small aliquot of resin was taken to evaluate the substitution degree of resin by Fmoc-test,^35^ resulting in 0.285 mmol/g. The synthesis was completed on a 0.05 mmol scale (175 mg of resin). The resin was swollen in DMF, Fmoc group removed, and the N-terminal amino acid *N*^α^-Boc-Orn(*N*^δ^-alloc,γ-mercapto-tBu)–OH (**10**) was coupled using 1.75 equiv of the amino acid, 1.68 equiv of HATU, and 3.5 equiv of DIPEA, for 1 h. The resin was washed with DMF, DCM, and diethyl ether and dried *in vacuo*.

#### Peptide Cleavage from the Resin and Deprotection

The dried resin (50 μmol, ∼175 mg) was transferred into a round-bottom glass flask containing a magnetic stirring bar and placed on ice. A solution of 2% thioanisole in TFA (3.5 mL) was added to the chilled resin and left under magnetic stirring for 10 min, on ice. Then 350 μL of TFMSA was added slowly and dropwise to the resin in the flask, and the reaction was left under vigorous stirring, on ice, for 30 min. The resin was removed by filtration and washed with TFA. The filtrates were added dropwise to cold diethyl ether (50 mL, ∼ten volume) to precipitate the crude peptide. The crude product, containing the peptide NH_2_–Orn (*N*^δ^-alloc,γ-mercapto,-Arg-Gly-Asp-d-Phe-*N-*Hnb-Cys-Gly-NH_2_) was recovered by centrifugation, solubilized in CH_3_CN/H_2_O 80/20, and lyophilized. 78 mg of crude peptide was obtained. The crude sample was analyzed by LC–MS (MW^th^ monoisotopic (with intramolecular S–S bond): 1031.643 Da); MW^exp^ monoisotopic: 1031.357 Da).

#### Peptide NH_2_–Orn(*N*^δ^-alloc,γ-mercapto)-Arg-Gly-Asp-d-Phe-*N-*Hnb-Cys-Gly-NH_2_ Head-to-Tail Cyclization via Intramolecular Native Chemical Ligation

The crude peptide (78 mg) was dissolved in 15 mL of NCL buffer 0.2 M sodium phosphate pH 7.1, 3.0 M guanidinium hydrochloride, 5.0 M imidazole, 25 mM TCEP, and incubated overnight (16 h) at room temperature under gentle stirring. The cyclization reaction mixture was analyzed by LC–MS and then purified by preparative reverse-phase HPLC, on an AXIA Kinetex 5 μm XB-C18 100 Å column 100 × 21.2 mm^2^, applying an isocratic phase of 3 min at 1% of CH_3_CN (0.1% TFA) in H_2_O (0.1% TFA) followed by a linear gradient of a gradient of CH_3_CN (0.1% TFA) in H_2_O (0.1% TFA) from 1% to 60% in 20 min, at a flow rate of 18 mL/min. 5 mL of NCL mixture was injected at each run, using a loop of 10 mL of volume. The eluted peak fractions were analyzed by LC–MS, and those containing pure cyclic peptide *cyclo*(-Orn(*N*^δ^-alloc,γ-mercapto)-Arg-Gly-Asp-d-Phe-) were pooled and lyophilized. 6.71 mg (7.52 μmol) of pure *cyclo*(-Orn(*N*^δ^-alloc,γ-mercapto)-Arg-Gly-Asp-d-Phe-) was obtained. Pure peptide sample was analyzed by LC-ESI ToF MS (MW^th^ monoisotopic:705. 470 Da; MW^exp^ monoisotopic: 705.293 Da).

#### In-Solution Allyloxycarbonyl (Alloc) Group Removal

2.8 mg (3.97 μmol) of *cyclo*(-Orn(*N*^δ^-alloc,γ-mercapto)-Arg-Gly-Asp-d-Phe-) was dissolved in 100 μL of dry DMF and diluted with 400 μL of dry DCM. 10 equiv of phenylsilane (40 μmol, 47.0 μL) and 0.175 equiv of tetrakistriphenylphosphine palladium (Pd(PPh_3_)_4_) (0.70 μmol, 0.80 mg) were added to the peptide solution. The reaction was incubated for 1 h, at room temperature, under stirring. DCM was removed under N_2_ flux, and the sample was diluted with 5 mL of H_2_O/TFA 0.1%. The sample was centrifuged, the supernatant recovered and purified by reverse-phase HPLC, on a Jupiter C18 5 μm 300 Å 250 × 10 mm^2^ column applying an isocratic phase of 10 min at 1% of CH_3_CN (0.1% TFA) in H_2_O (0.1% TFA) followed by a linear gradient of CH_3_CN (0.1% TFA) in H_2_O (0.1% TFA) from 1–60% in 20 min, at a flow rate of 5 mL/min. Peak fractions were analyzed by LC-ESI ToF MS, and those containing pure *cyclo*(-Orn(γ-mercapto)-Arg-Gly-Asp-d-Phe-) as monomer or as disulfide bonded dimer were collected and lyophilized. 1.0 mg (1.61 μmol) of pure *cyclo*(-Orn(γ-mercapto)-Arg-Gly-Asp-d-Phe-) was obtained (yield of 40.3%). Pure peptide sample was analyzed by LC-ESI ToF MS before and after treatment with TCEP 10 mM (MW^th^ monoisotopic monomer: 621.389 Da; MW^exp^ monoisotopic monomer: 621. 271 Da; MW^th^ monoisotopic dimer: 1240.762 Da; MW^exp^ monoisotopic dimer: 1240.530 Da).

#### Synthesis of *cyclo*(-Orn(*N*^δ^-CO-C4-AAZTA,γ-mercapto)-Arg-Gly-Asp-d-Phe-) via Native Chemical Ligation

2.24 mg (3.61 μmol, 1.0 equiv) of cyclo(-Orn(γ-mercapto)-Arg-Gly-Asp-d-Phe-) was dissolved in 1.5 mL (peptide final concentration 2.4 mM) of native chemical ligation buffer containing buffer 0.2 M sodium phosphate pH 7.1, 3.0 M guanidinium hydrochloride, 5.0 M imidazole, 25 mM TCEP, 2 mM ethylenediaminetetraacetic acid (EDTA), in ultrapure water. The sample mixture was left for 30 min at room temperature to allow the reduction of thiol groups, then 3.0 mg (5.42 μmol, 1.5 equiv) of AAZTA-C4-CO-MES was added to the reaction mixture (final concentration 3.5 mM) and gently stirred until complete solubilization was observed. The reaction was incubated at room temperature, under mild stirring, overnight (16 h). The native chemical ligation reaction mixture was analyzed by LC-ESI ToF MS and then purified by preparative reverse-phase HPLC, on a Jupiter C18 5 μm 300 Å 250 × 10 mm^2^, with an applied isocratic phase of 10 min of 1% CH_3_CN (0.1% TFA) in H_2_O (0.1% TFA) followed by a linear gradient from 1–60% in 20 min, at a flow rate of 5 mL/min. Eluted peak fractions were analyzed by LC-ESI ToF MS. Pure fractions of *cyclo*(-Orn(*N*^δ^-CO-C4-AAZTA,γ-mercapto)-Arg-Gly-Asp-d-Phe-) were collected and lyophilized. 2.31 mg (2.21 μmol) of pure *cyclo*(-Orn(*N*^δ^-CO-C4-AAZTA,γ-mercapto)-Arg-Gly-Asp-d-Phe-) was obtained (yield of 61.2%). Pure product was characterized by LC-ESI ToF MS (MW^th^ monoisotopic: 1049.811 Da; MW^exp^ monoisotopic: 1103.351 Da (iron adduct)).

#### Synthesis of *cyclo*(-Orn(*N*^δ^-CO-C4-AAZTA,γ-*S*-succinimido-Cy5.5)-Arg-Gly-Asp-d-Phe-) via Thiol-maleimide Addition

1.2 mg (1.14 μmol, 1.0 equiv) of *cyclo*(-*N*^δ^-AAZTA-C4-CO-Orn(γ-mercapto)-Arg-Gly-Asp-d-Phe-) was dissolved in 1 mL of labeling buffer, 20 mM sodium phosphate pH 7.4 (peptide concentration 1.1 mM). 1.2 mg (1.71 μmol, 1.5 equiv) of the cyanine 5.5 maleimide was dissolved in 200 μL of ultrapure CH_3_CN (8.5 mM). The nominal concentration of the cyanine 5.5 maleimide was confirmed by UV–vis spectroscopy, evaluating the absorbance at 684 nm of a 1:5000 dilution of the dye solution in CH_3_CN/H_2_O 80/20, using the cyanine 5.5 molar extinction coefficient at 684 nm of 1.98 × 10^5^ M^–1^cm^–1^ (https://www.lumiprobe.com/p/cy55-maleimide). The dye solution was added to the peptide dissolved in labeling buffer, and the reaction was allowed to proceed at room temperature, for 3 h, in the dark. The reaction mixture was analyzed by LC-ESI ToF MS performed on a LUNA Omega Polar C18 3 μm 300 Å 100 × 2.1 mm^2^ column (Phenomenex), applying a linear gradient of gradient of CH_3_CN (0.1% TFA) in H_2_O (0.1% TFA) from 5–70% in 20 min, at a flow rate of 0.2 mL/min. The reaction mixture was diluted to 5 mL with H_2_O (0.1% TFA) and purified by preparative reverse-phase HPLC on a Jupiter C18 5 μm 250 × 10 mm^2^, 300 Å (Phenomenex), applying an isocratic phase of 5 min at 5% of CH_3_CN (0.1% TFA) in H_2_O (0.1% TFA) followed by a linear gradient from 5–70% in 20 min, at a flow rate of 5 mL/min. Eluted peak fractions were analyzed by LC-ESI ToF MS. Pure fractions of *cyclo*(-Orn(*N*^δ^-CO-C4-AAZTA,γ-*S*-succinimido-Cy5.5)-Arg-Gly-Asp-d-Phe-) were collected and lyophilized. 1.29 mg (735 nmol) of pure *cyclo*(-Orn(*N*^δ^-CO-C4-AAZTA,γ-*S*-succinimido-Cy5.5)-Arg-Gly-Asp-d-Phe-) was obtained (yield of 64.5%). Pure product was characterized by LC-ESI ToF MS using a LUNA Omega Polar C18 3 μm 300 Å 100 × 2.1 mm^2^ column (Phenomenex), applying a linear gradient of gradient of CH_3_CN (0.1% TFA) in H_2_O (0.1% TFA) from 5–70% in 20 min, at a flow rate of 0.2 mL/min (MW^th^ monoisotopic monomer: 1755.717 Da; MW^exp^ monoisotopic: 1755.809 and 1808.720 Da (Fe(III) adduct)).

### Complexation with Gallium

0.54 mg of *cyclo*(-Orn(*N*^*δ*^-CO-C4-AAZTA,γ-*S*-succinimido-Cy5.5)-Arg-Gly-Asp-d-Phe-) (0.3 μmol) was dissolved in 450 μL of a solution 50:50 of 0.1 M acetate buffer pH = 4.6/CH_3_CN. Under stirring, an equimolar amount of GaCl_3_ (50 μL of a 6.0 M standard water solution) was added and the solution was stirred at room temperature for 1 h. The Ga-complex was analyzed by direct infusion ESI (+) on a Waters ACQUITY QDa Detector (MW^th^ average 1823.76 Da; MW^exp^: average 1.823.21 Da).

### *In Vitro* Receptor Binding Analysis by Flow Cytometry

Human U-87 MG glioblastoma cells were used to determine cell binding of Gallium labeled cyclo(-Orn(*N*^*δ*^-CO-C4-AAZTA,γ-*S*-succinimido-Cy5.5)-Arg-Gly-Asp-d-Phe-). To minimize the nonspecific uptake, incubations were performed on ice and followed immediately by flow cytometry. All cell groups (10^5^) were incubated with Gallium labeled *cyclo*(-Orn(*N*^*δ*^-CO-C4-AAZTA,γ-*S*-succinimido-Cy5.5)-Arg-Gly-Asp-d-Phe-) for 1 h on ice with the probe 0.2, 0.4, 0.8, and 2.3 μM. After centrifugation of the tubes and the elimination of the supernatant, cells were washed twice with PBS 1X. 100 μL of PBS 1X supplemented with 0.1% BSA was added to each tube.

## Data Availability

The data underlying this study are available in the published article and its Supporting Information.

## Supporting Information Available

Synthesis scheme of compound **10**, additional experimental NMR and LC-mass spectra, UV–vis spectrum of gallium complexed peptide probe (PDF)
