# Supplementary material for: A Chemical Strategy for the Preparation of Multimodified Peptide Imaging Probes
Source: J Org Chem. 2023 Mar 29;88(7):4546–53. doi: 10.1021/acs.joc.3c00014 (PMC10088022; doi:10.1021/acs.joc.3c00014)
Supplement: Supplementary file 1 — jo3c00014_si_001.pdf [file jo3c00014_si_001.pdf]

Supporting Information

A chemical strategy for the preparation of multi-modified peptide imaging probes

Lucia De Rosa [§‡], Ivan Hawala [¥†‡], Rossella Di Stasi [§], Rachele Stefania [¥‡], Martina Capozza [¥], Donatella Nava [¶], Luca Domenico D'Andrea [#*]

[§] Istituto di Biostrutture e Bioimmagini, Consiglio Nazionale Delle Ricerche, Via Pietro Castellino 111, 80131, Napoli - Italy;

[¥] Centro di Imaging Molecolare, Dipartimento di Biotecnologie Molecolari e Scienze per La Salute, Università di Torino, via Nizza 52, 10126, Torino - Italy;

[¶] Dipartimento di Scienze Farmaceutiche, Università di Milano, Via Venezian 21, 20133 Milano - Italy

[#] Istituto di Scienze e Tecnologie Chimiche "G. Natta", Consiglio Nazionale Delle Ricerche, Via M. Bianco 9, 20131, Milano - Italy.

List of Contents

Supplementary figures

- Scheme S1. Synthesis scheme of ***10***
- Figure S1. ^{1}H NMR spectrum ***1***
- Figure S2. ^{1}H NMR spectrum ***2***
- Figure S3. ^{1}H NMR spectrum of ***3***
- Figure S4. ^{1}H NMR spectrum of ***4***
- Figure S5. ^{1}H NMR spectrum and LC-MS analysis of ***5***
- Figure S6. ^{1}H NMR spectrum and LC-MS analysis of pure compound of ***6***
- Figure S7. ^{1}H NMR spectrum of and LC-MS analysis of pure compound ***8***
- Figure S8. ^{1}H NMR spectrum of and LC-MS analysis of pure compound ***9***
- Figure S9. Bidimensional characterization of compound ***10***.
- Figure S10. ^{1}H and ^{13}C NMR spectra, HPLC profile revealed at 210 nm and ESI mass spectrum of ***10***
- Fig. S11. Analysis by LC-MS of pure *cyclo*(-Orn(N^{δ}-alloc, γ-mercapto)-Arg-Gly-Asp-D-Phe-)
- Fig. S12. Analysis by LC-MS of pure cyclo(-Orn(γ-mercapto)-Arg-Gly-Asp-D-Phe-)
- Fig. S13. Analysis by LC-MS of pure *cyclo*(-Orn(N^{δ}-CO-C4-AAZTA,γ-mercapto)-Arg-Gly-Asp-D-Phe-)
- Fig. S14. LC-MS analysis of the Ga(III)-complexed *cyclo*(-Orn(N^{δ}-CO-C4-AAZTA,γ-S-succinimido-Cy5.5)-Arg-Gly-Asp-D-Phe-)
- Fig. S15. UV-vis spectrum of *cyclo*(-Orn(N^{δ}-CO-C4-AAZTA,γ-S-succinimido-Cy5.5)-Arg-Gly-Asp-D-Phe-) complexed with Ga(III)

Supplementary figures

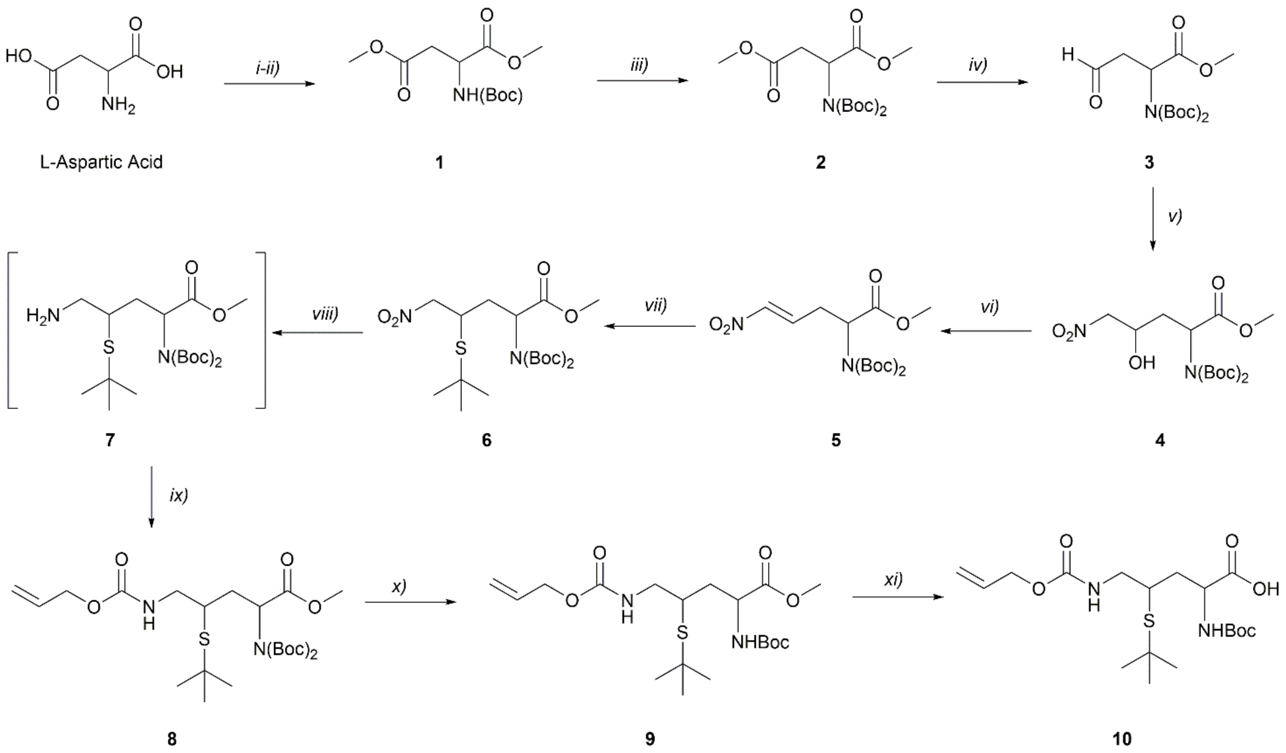

Scheme S1. Synthesis scheme of ***10***: *i)*Me_3SiCl, MeOH; *ii)*$(Boc)_2O$, Et_3N; *iii)* Boc_2O, DMAP (4-dimethylaminopyridine), CH_3CN; *iv)* DIBAL (diisobutyalluminium hydrate), Et_2O; *v)* CH_3NO_2, TBAF (tetra N-butylammonium fluoride); *vi)*Ac_2O, DMAP, Et_2O; *vii)t*Bu-SH, *n*BuLi, THF; *viii)* $NaBH_4$, $NiCl_2$, MeOH/THF; *ix)* Allyl Chloroformate, TEA (triethylamine), THF; *x)* TFA/CH_2Cl_2, 0 °C, 1 h, Boc_2O; *xi)* NaOH, *i*Pro/H_2O.

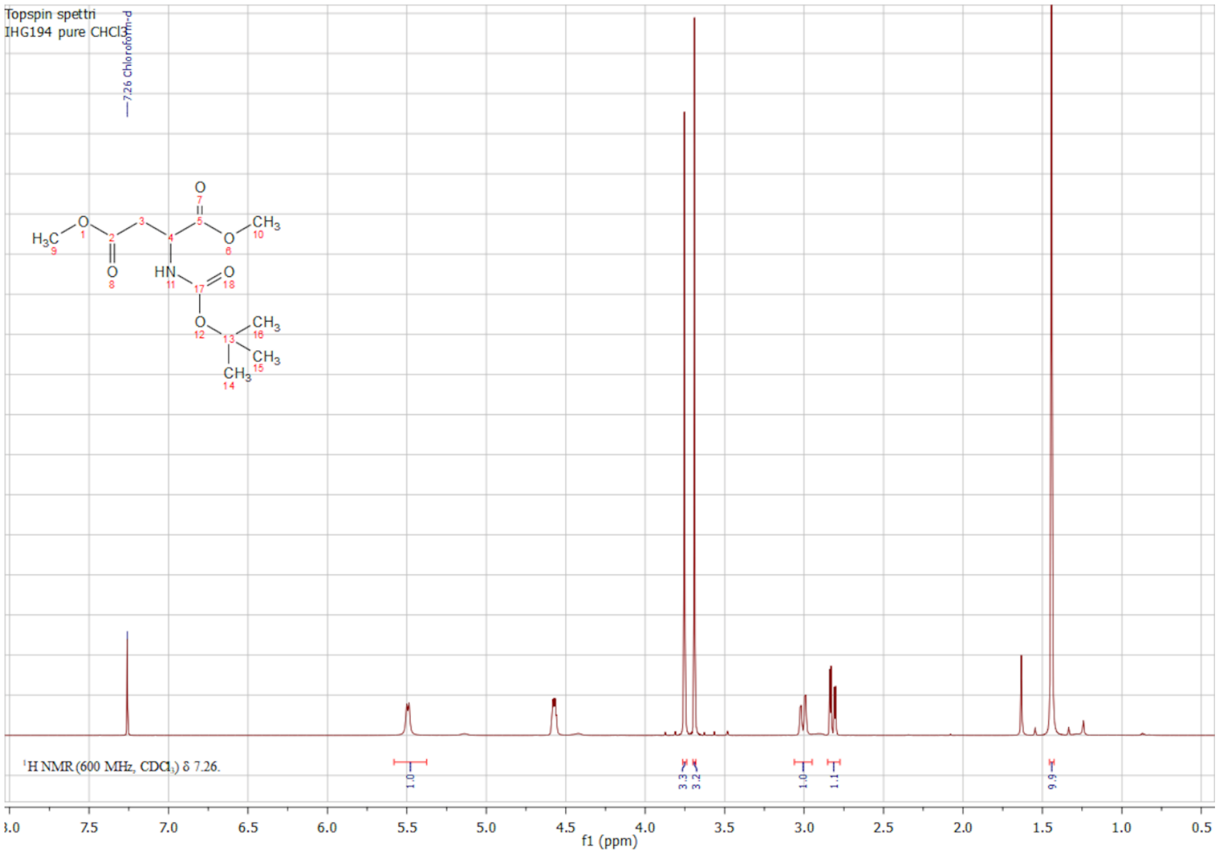

Figure S1. ^{1}H NMR spectrum of dimethyl (S)-2-tert-butoxycarbonylamino-butanodioate (***1***).

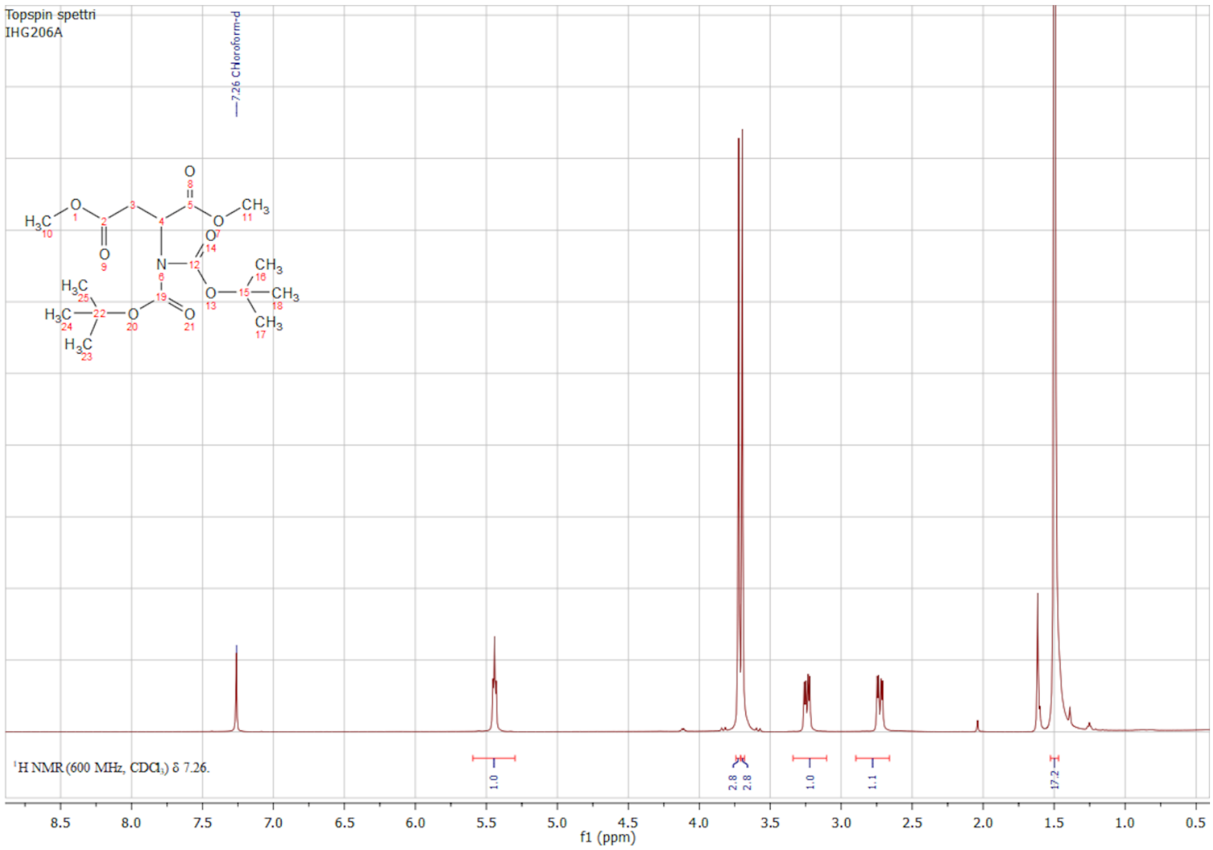

Figure S2. ^{1}H NMR spectrum of dimethyl (2S)-2-{(tert-butoxy)-N-[(tert-butyl)oxycarbonyl]carbonylamino}butane-1,4-dioate (***2***).

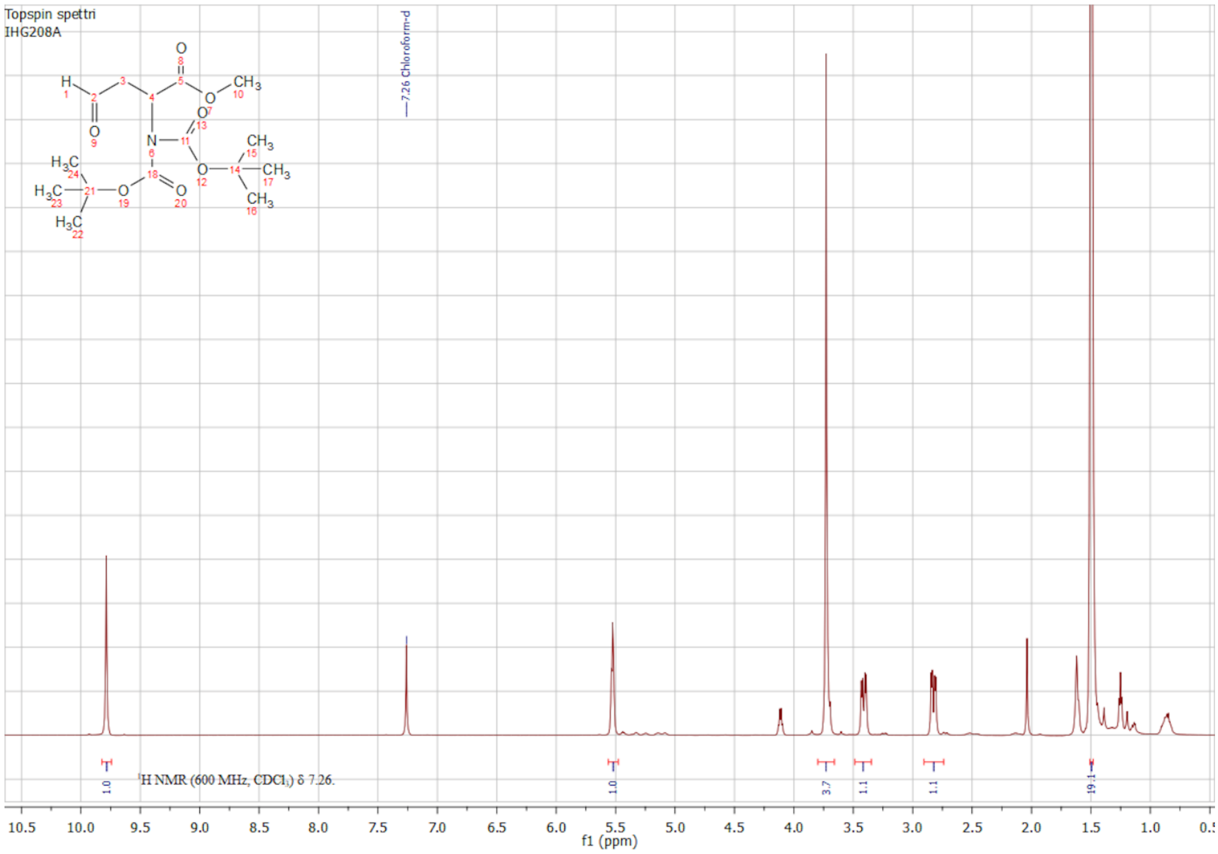

Figure S3. ^{1}H NMR spectrum of methyl (2S)-2-{(tert-butoxy)-N-[(tert-butyl)oxycarbonyl]carbonylamino}-4 oxobutanoate (***3***).

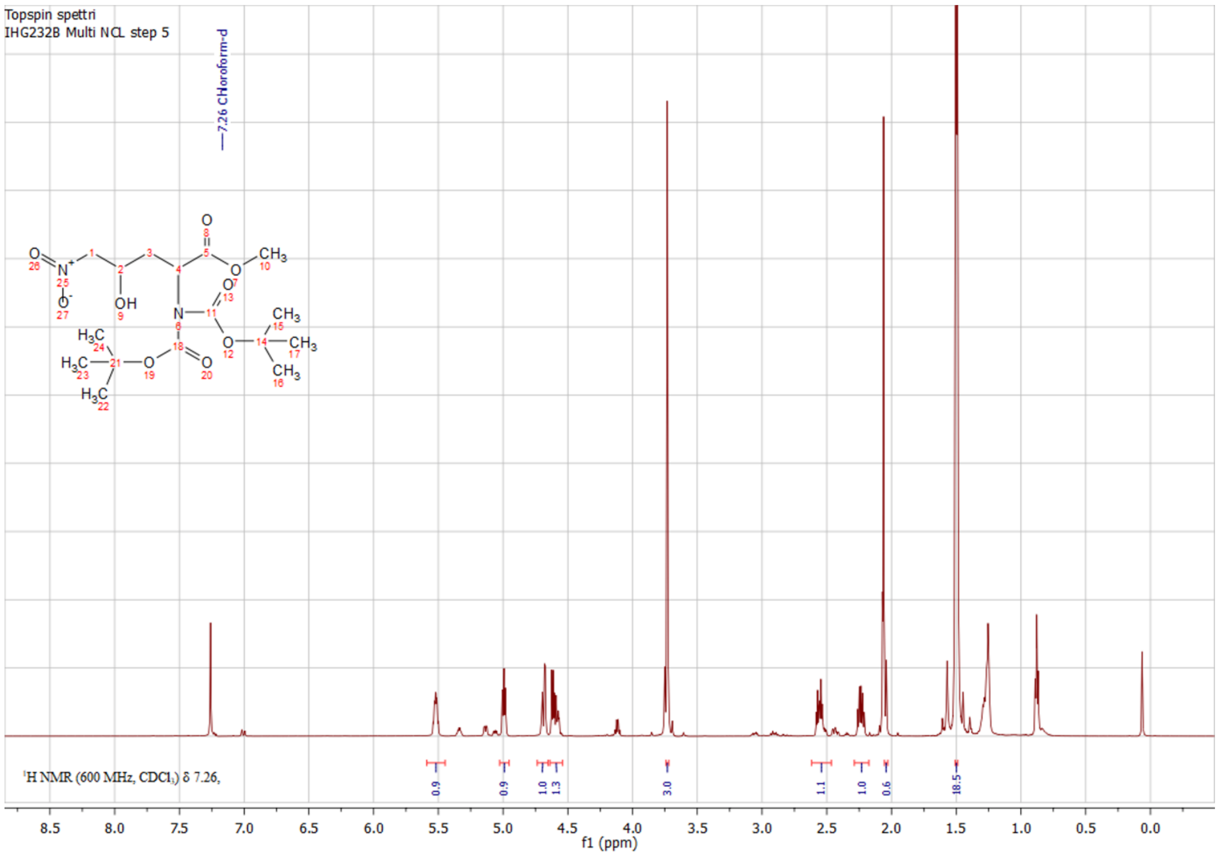

Figure S4. ^{1}H NMR spectrum of methyl (5R,S)-hydroxy-5-nitro-(2S)-2-{(tert-butoxy)-N-[tert-butyloxy carbonyl]-carbonylamino}-pentanoate (***4***).

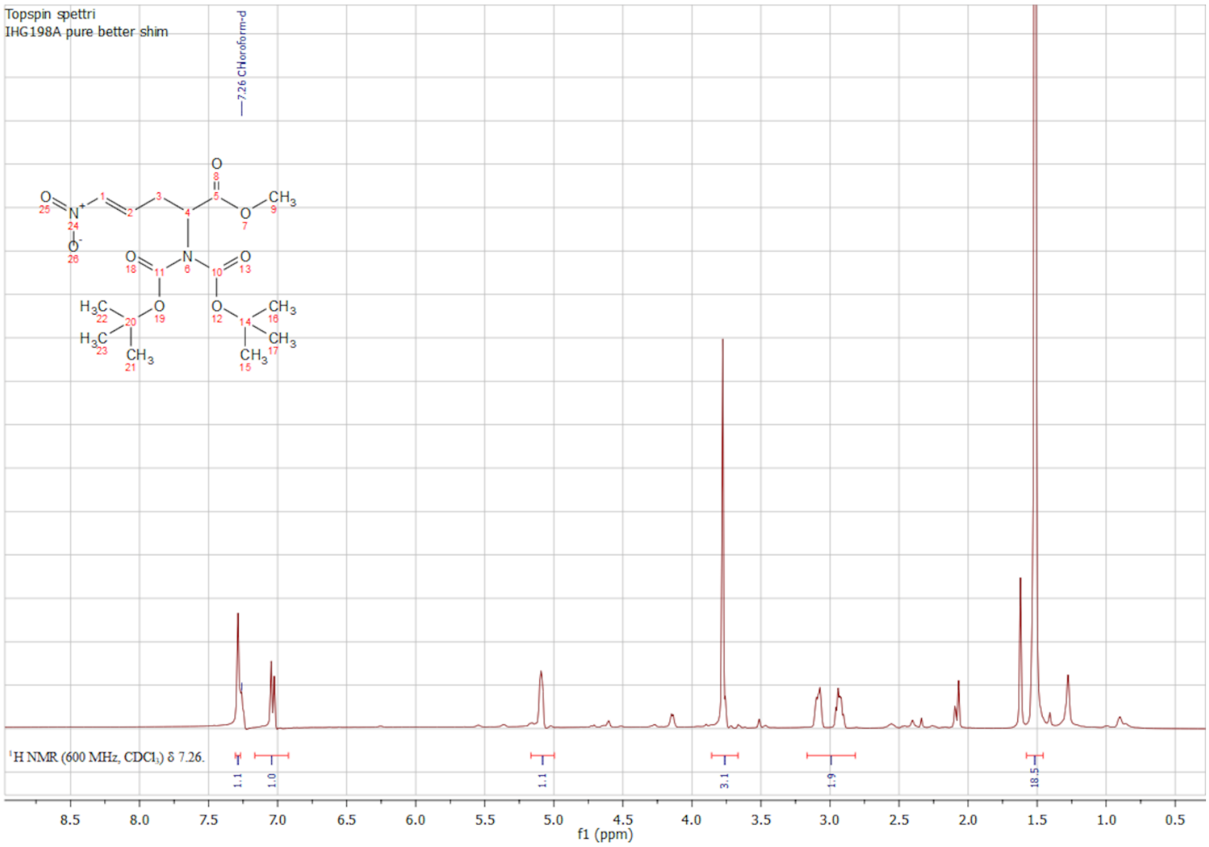

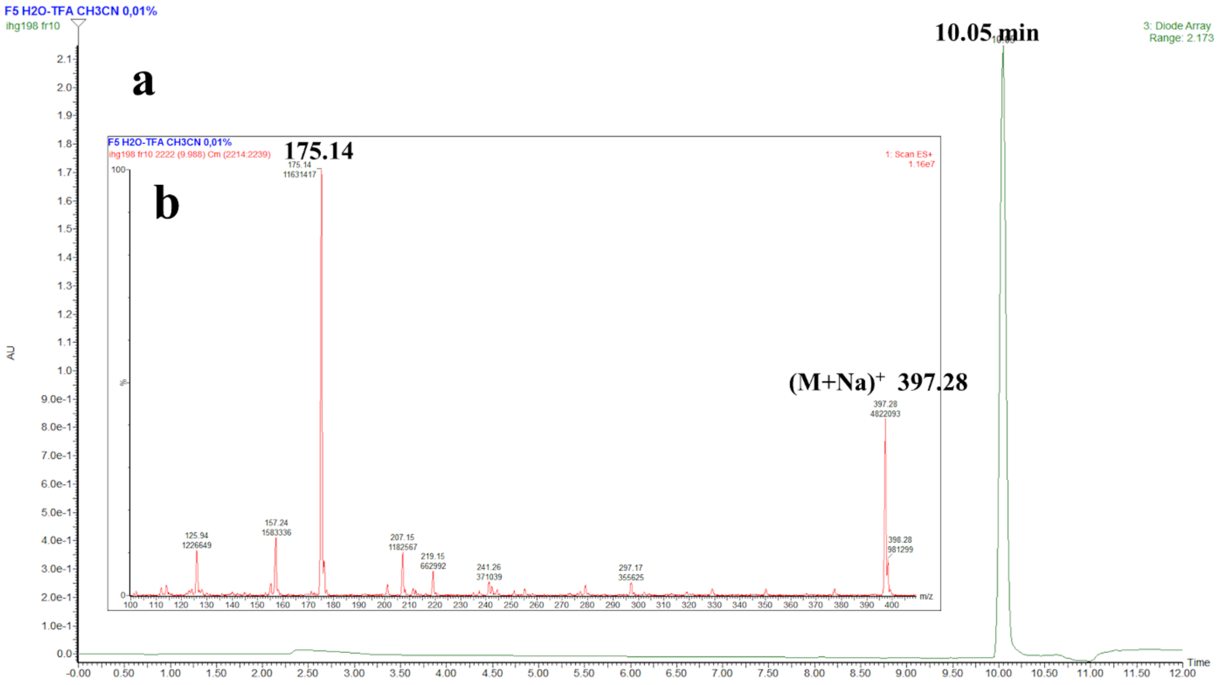

Figure S5. Top: ^{1}H NMR spectrum of methyl 5-nitro-2-(bis(tert-butoxycarbonyl)amino)-pent-4-enoate (***5***); bottom: LC-MS analysis of pure compound ***5***, a) chromatographic profile of ***5*** revealed at 210 nm and ESI-MS spectrum (b) of the peak at 10.05 min retention time.

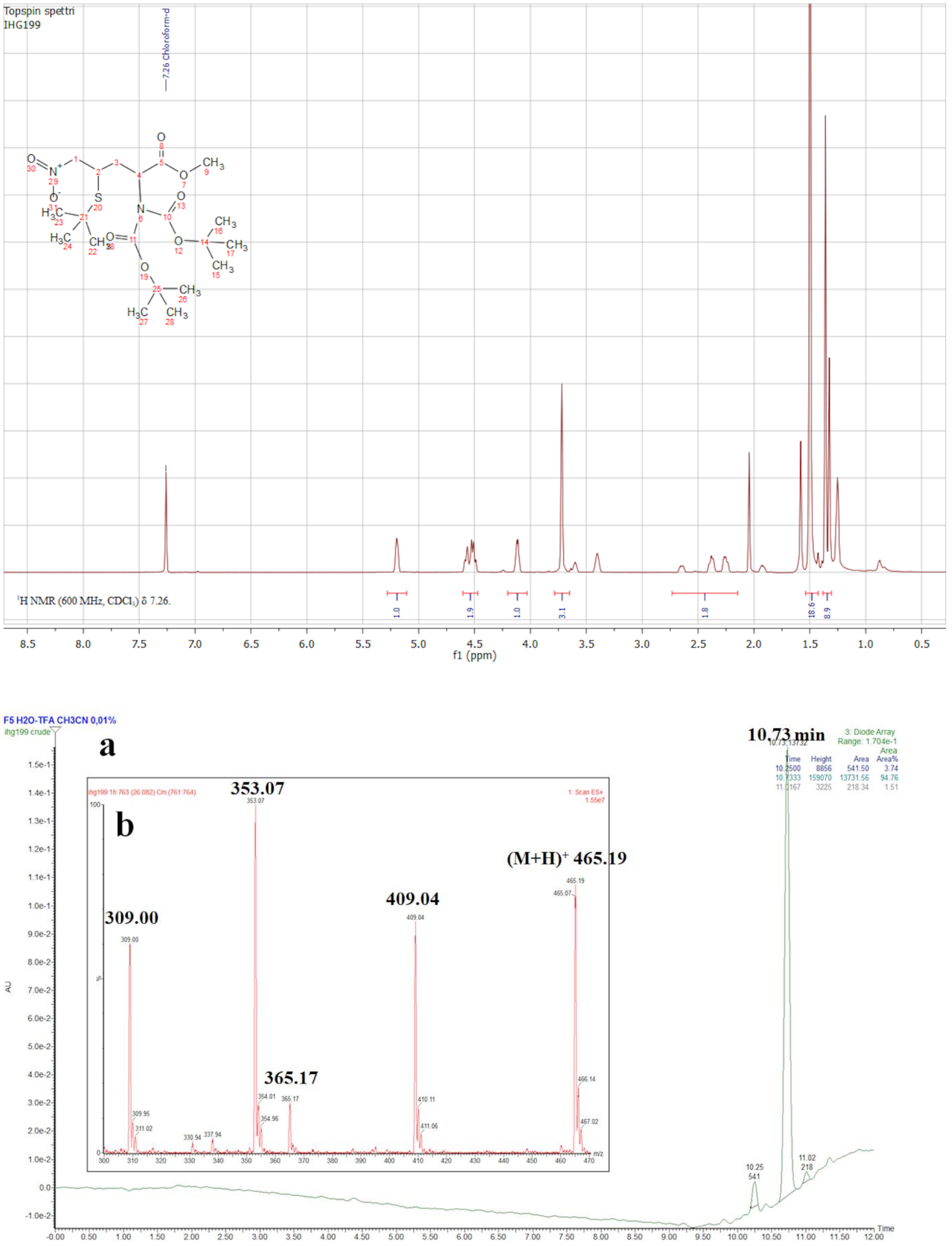

Figure S6. Top: ^{1}H NMR spectrum of methyl 5-nitro-2-(bis(tert-butoxycarbonyl)amino)-4-(tert-butylthio)pentanoate (*6*); bottom: LC-MS analysis of pure compound ***6***, a) chromatographic profile of **6** revealed at 210 nm and ESI-MS spectrum (b) of the peak at 10.73 min retention time.

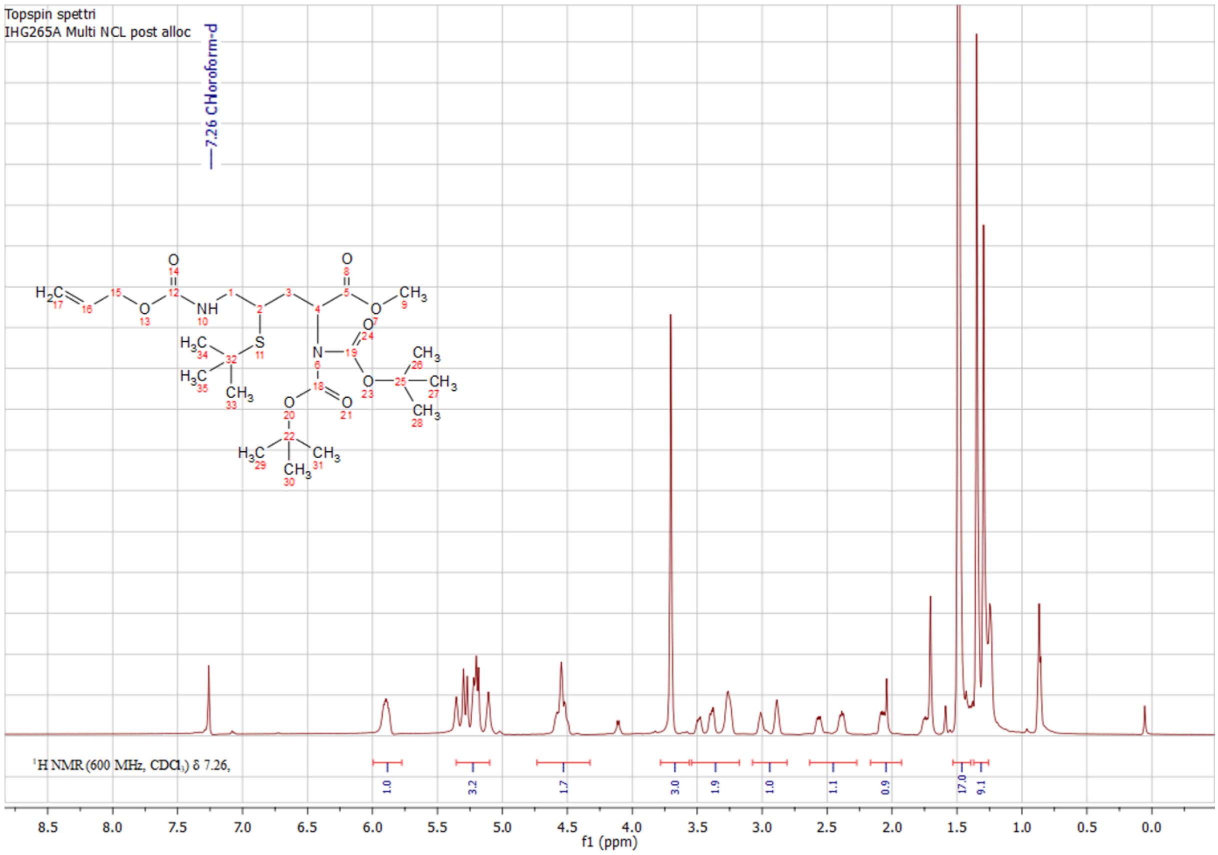

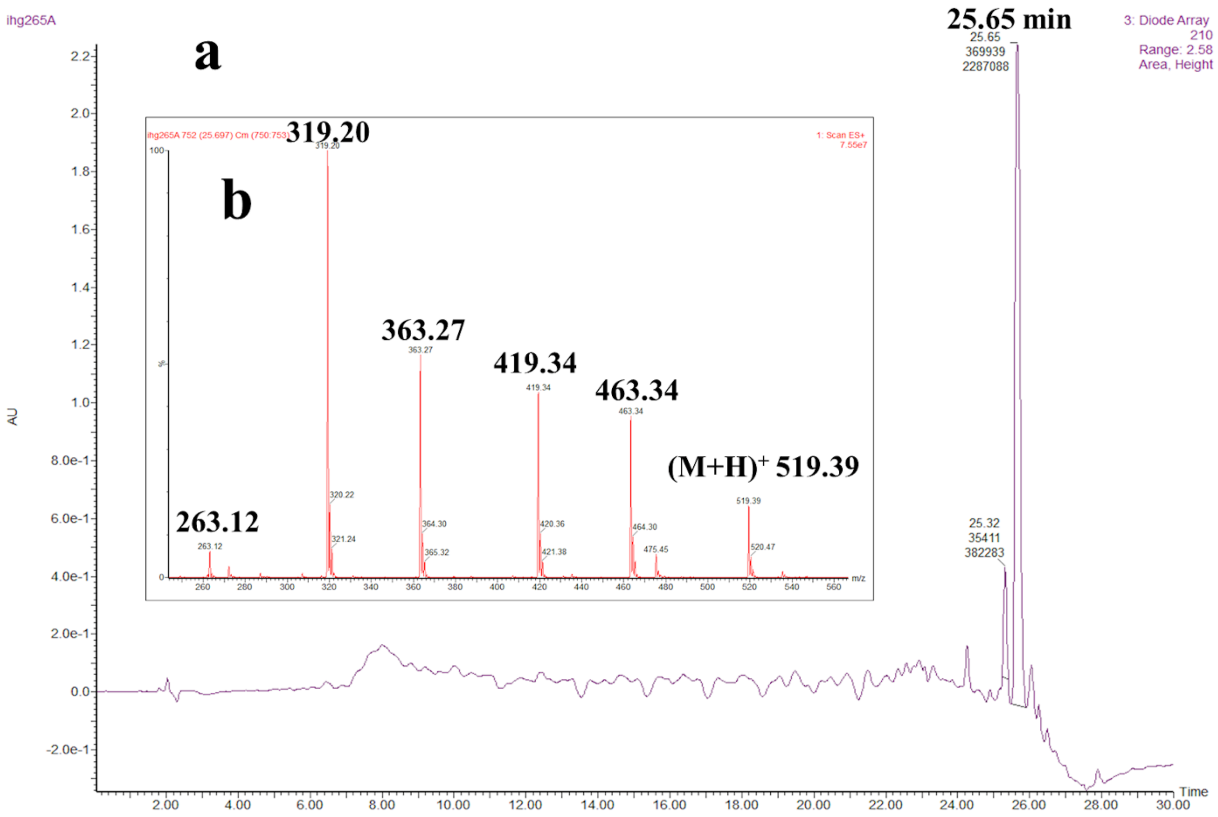

Figure S7. Top: ^{1}H NMR spectrum of methyl 5-(((allyloxy)carbonyl)amino)-2-(bis(tert-butoxycarbonyl)amino)-4-(tert-butylthio)pentanoate (***8***); bottom: LC-MS analysis of pure compound

8, a) chromatographic profile of ***8*** revealed at 210 nm and ESI-MS spectrum (b) of the peak at 25.65 min retention time.

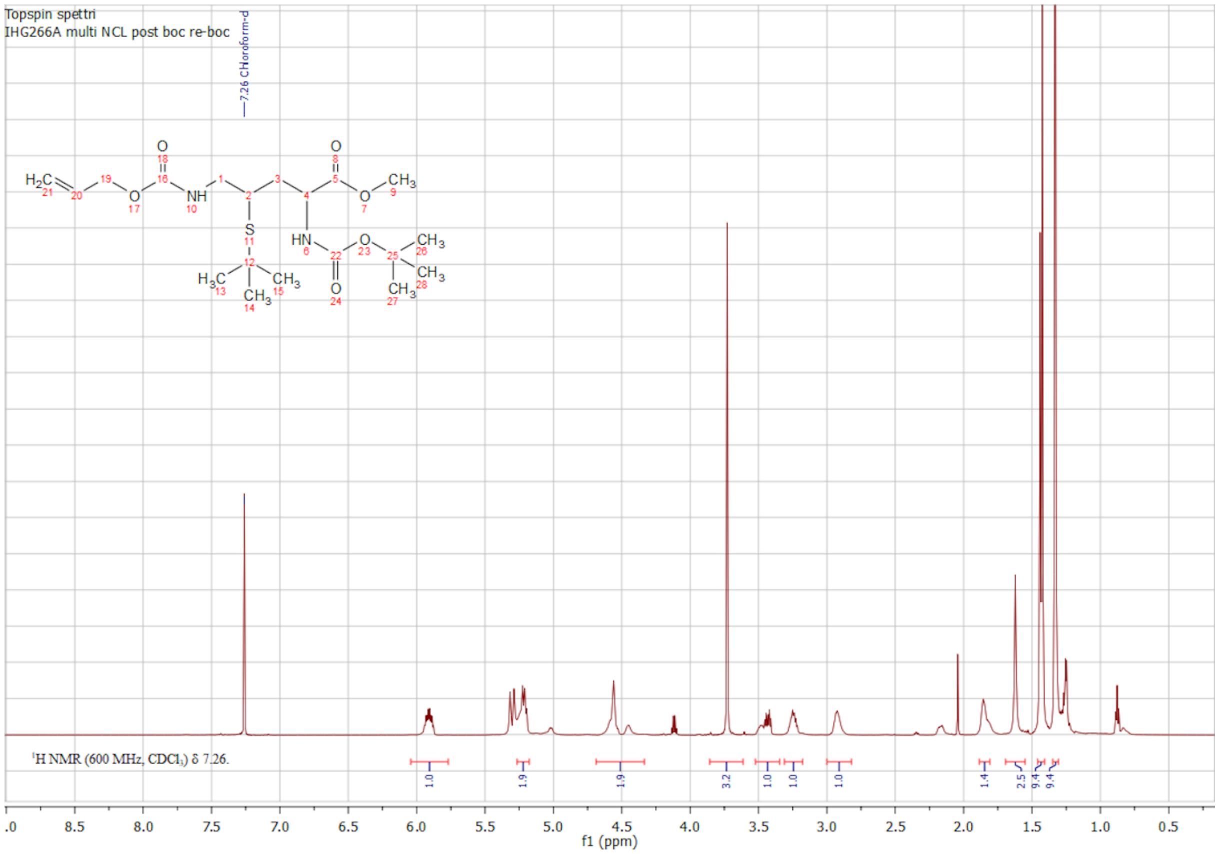

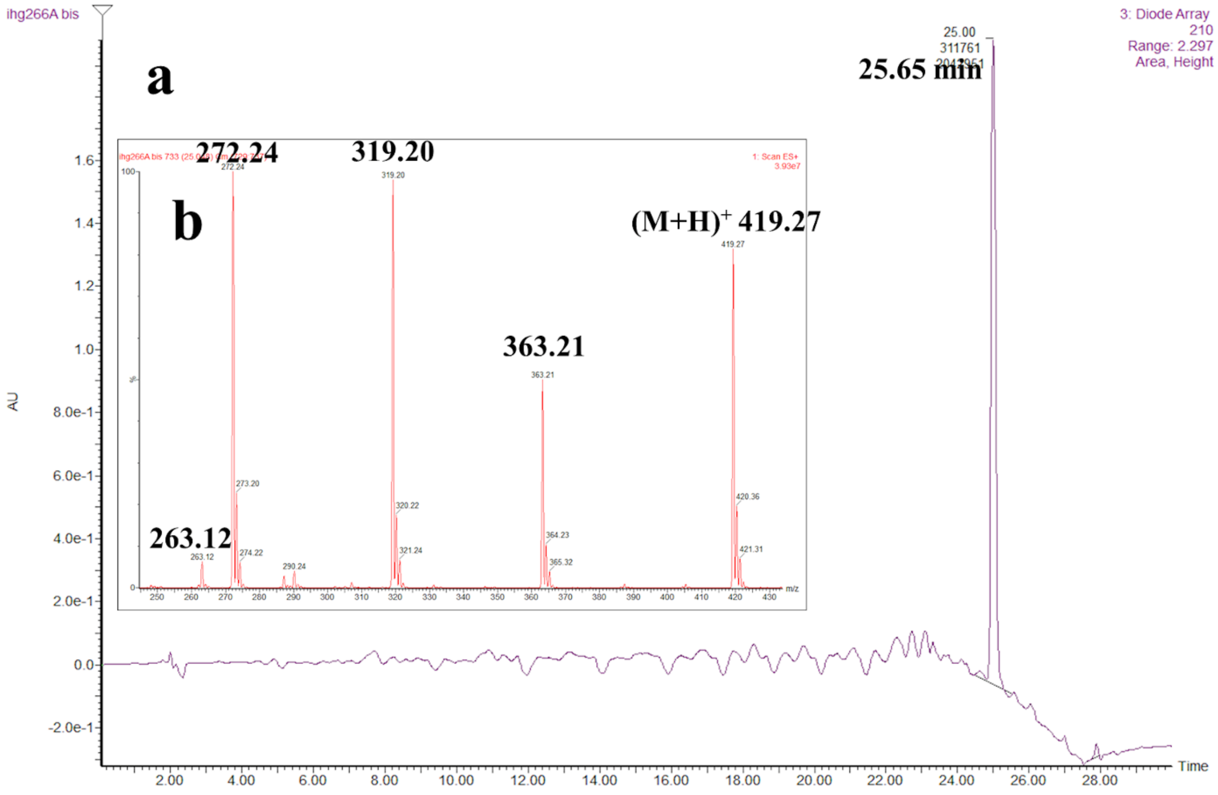

Figure S8. Top: ¹H NMR spectrum of methyl 5-(((allyloxy)carbonyl)amino)-2-((tert-butoxycarbonyl)amino)-4-(tert-butylthio)pentanoate (***9***); bottom: LC-MS analysis of pure compound

9, a) chromatographic profile of ***9*** revealed at 210 nm and ESI-MS spectrum (b) of the peak at 25.00 min retention time.

COSY in CDCl3 a T=300K , 400MHz

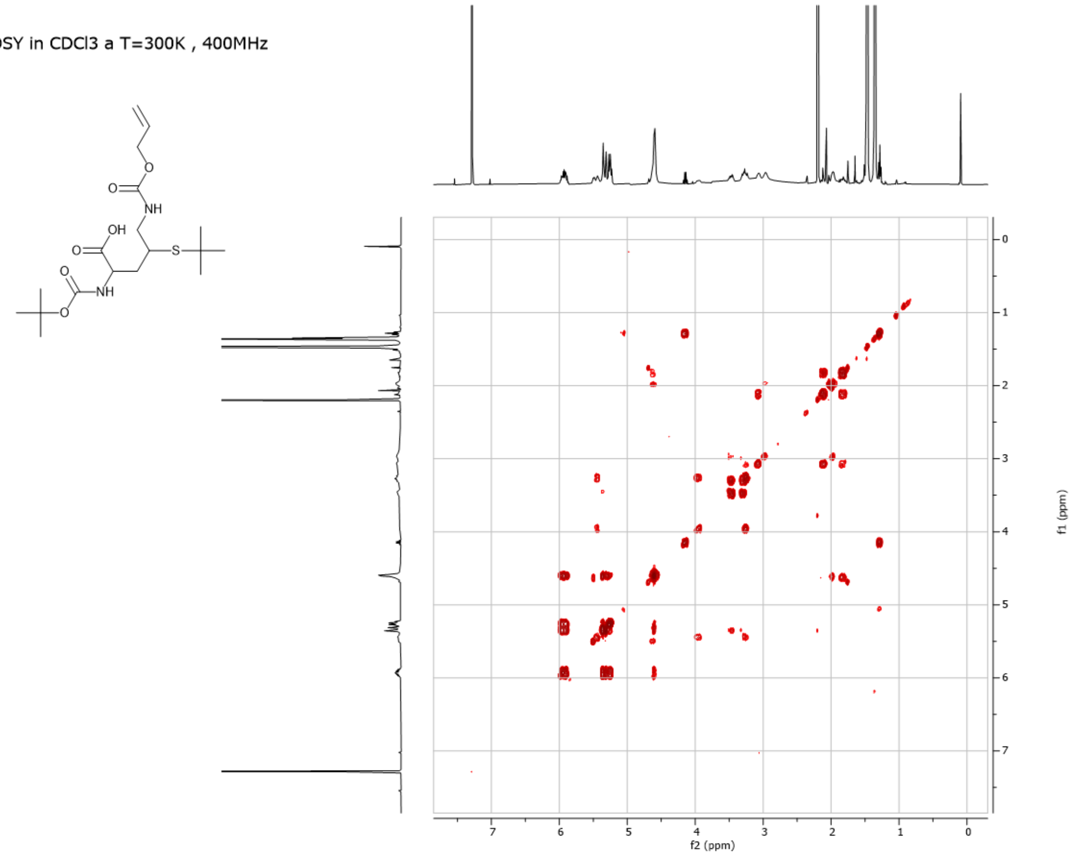

TOCSY in CDCl3 a T=300K (mixing time=60ms), 400MHz

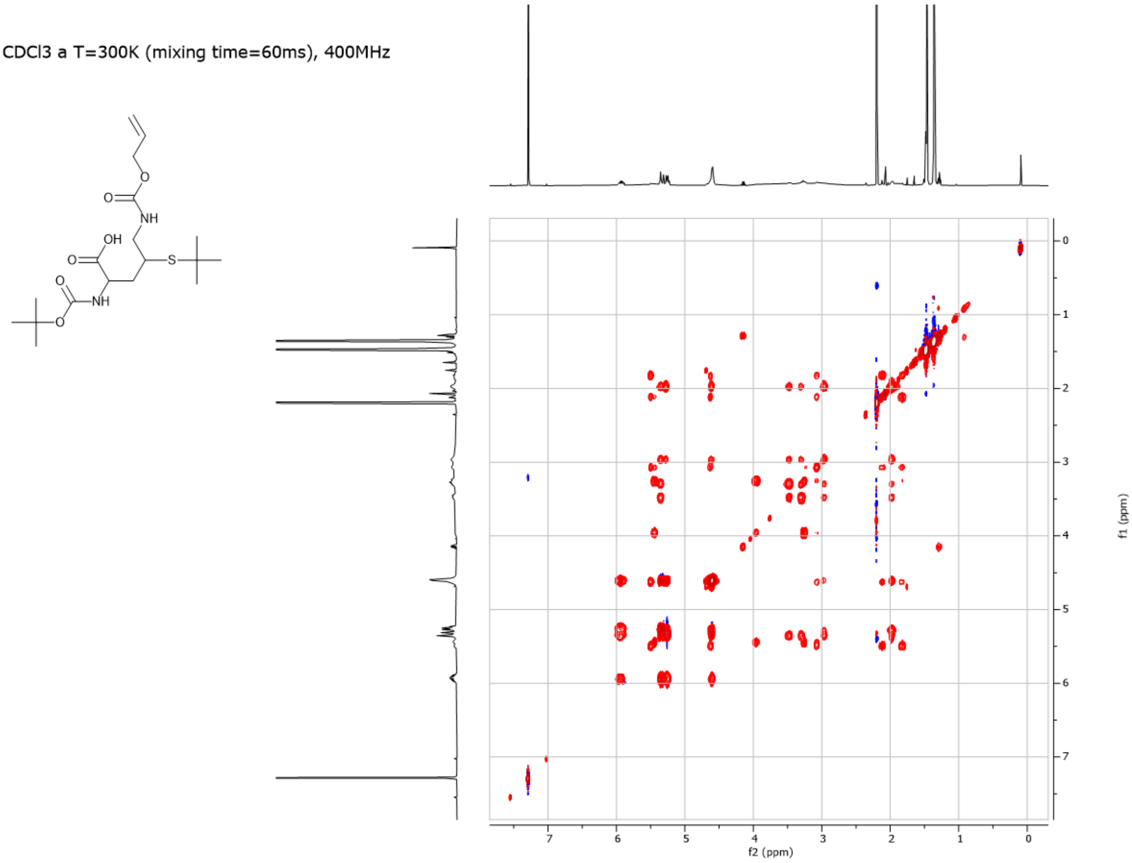

HSQC in CDCl3 a T=300K , 400MHz

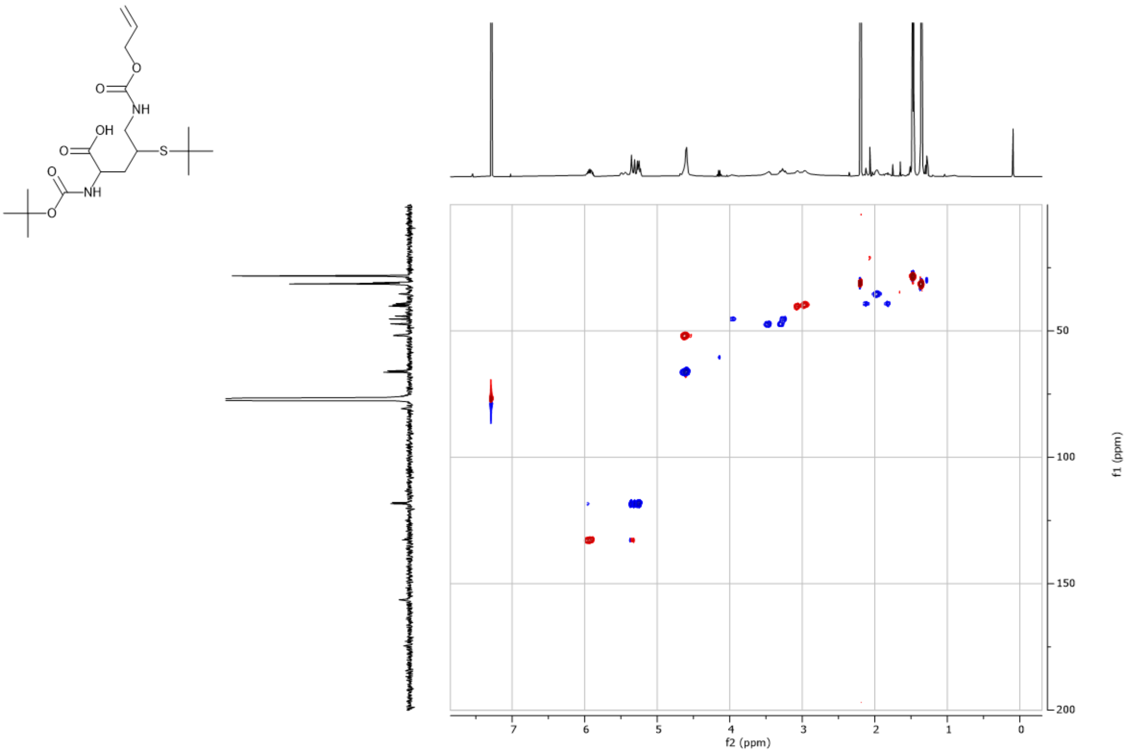

Figure S9. Bidimensional characterization of compound #10.

a

1H NMR (CDCl3 , 400MHz)

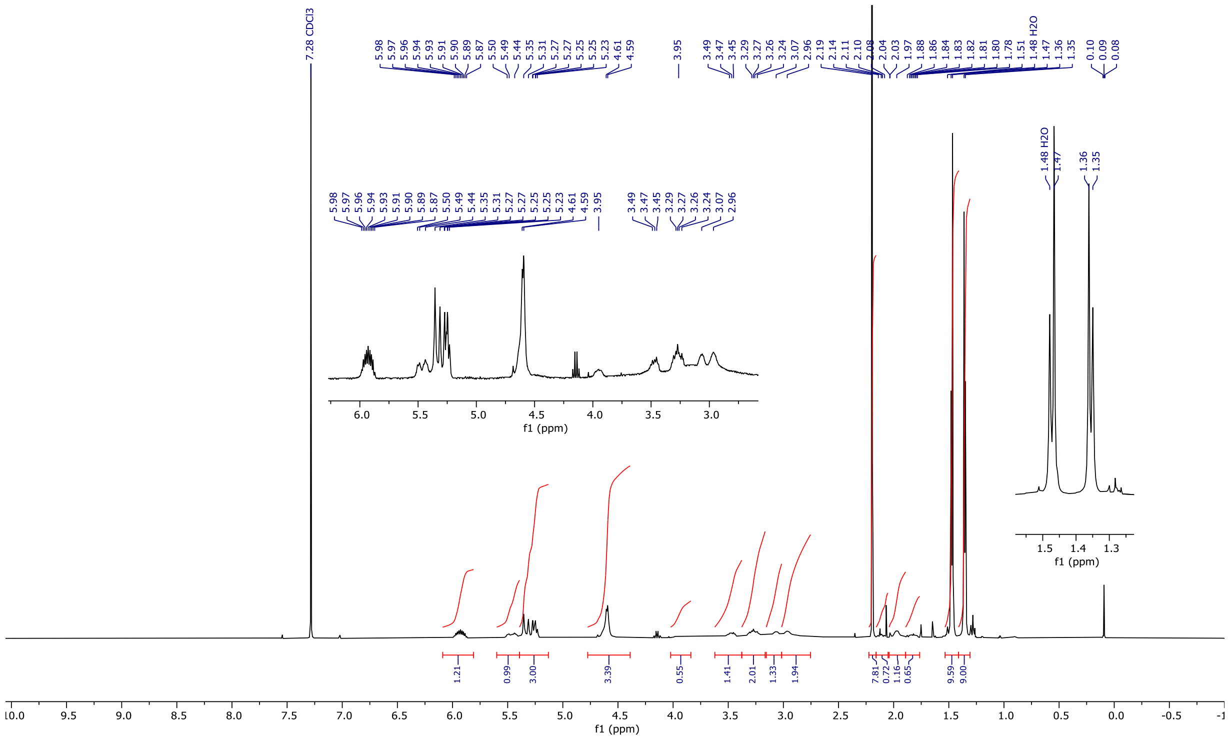

$^{13}C\{^1H\}$ NMR ($CDCl_3$, 100 MHz)

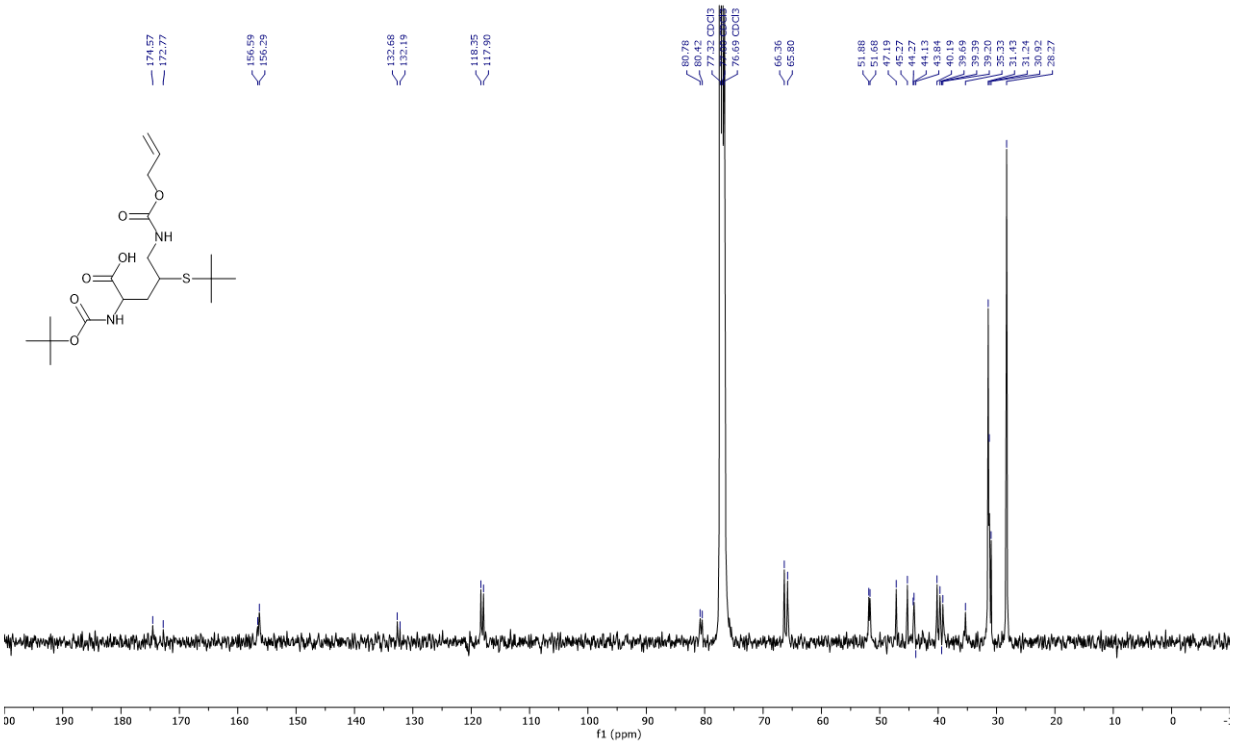

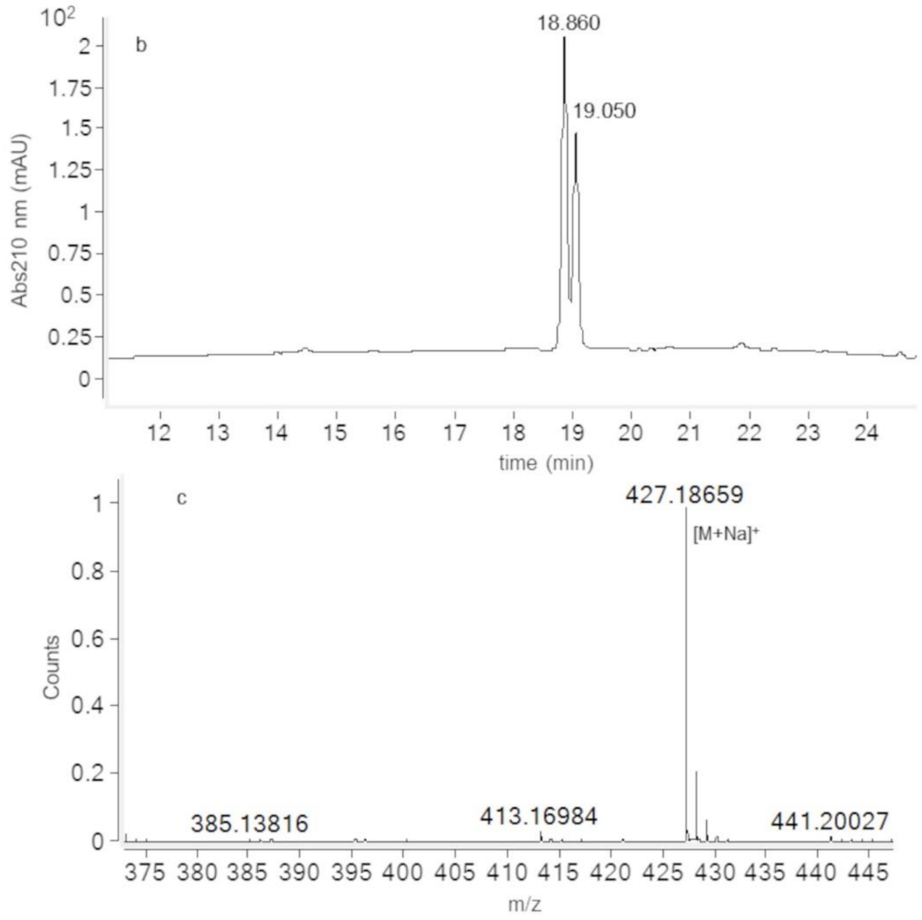

Figure S10. ^{1}H and ^{13}C{^{1}H} NMR spectra (a) in $CDCl_3$, HPLC profile revealed at 210 nm (b) and ESI mass spectrum (c) of 5-(((allyloxy)carbonyl)amino)-2-((tert-butoxycarbonyl)amino)-4-(tert-butylthio)pentanoic acid (***10***). HRMS (ESI/TOF) m/z: $[M + Na]^+$ Calcd for $C_{18}H_{32}N_2O_6SNa$ 427.1981; found 427.1866.

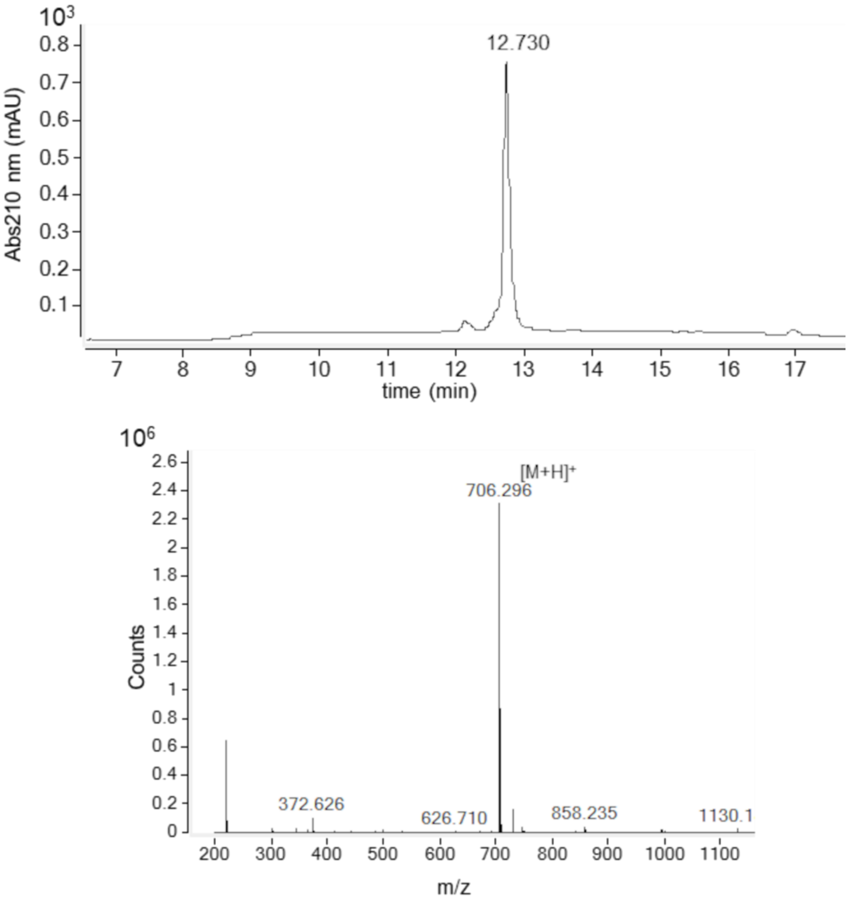

Figure S11. Analysis by LC-MS of pure *cyclo*(-Orn(N^{δ}-alloc, γ-mercapto)-Arg-Gly-Asp-D-Phe-) peptide obtained after intramolecular cyclization by native chemical ligation.

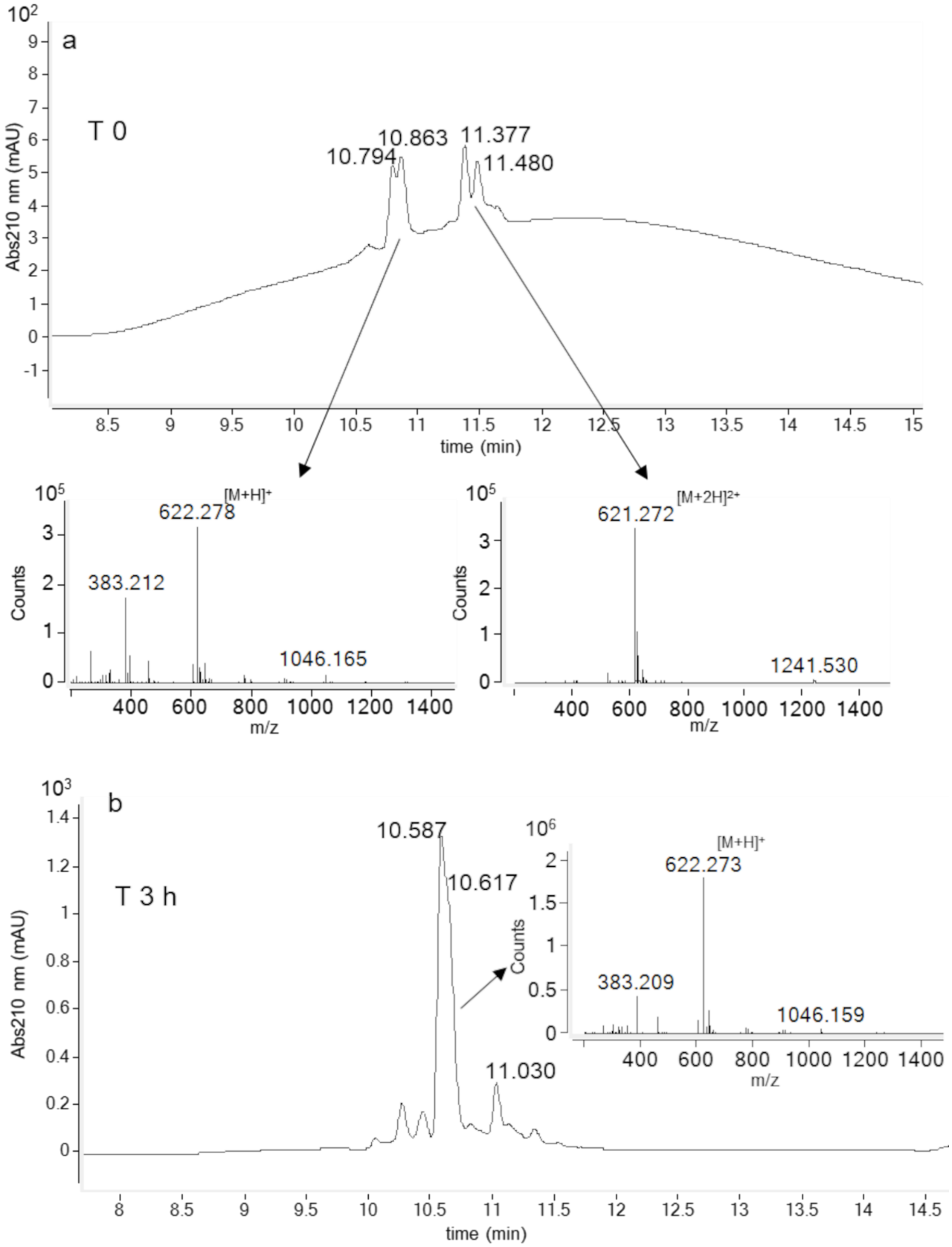

Figure S12. Analysis by LC-MS of pure fully unprotected *cyclo*(-Orn(γ-mercapto)-Arg-Gly-Asp-D-Phe-) obtained after Alloc removal. T 0 (a) and T 3 h (b) of incubation in TCEP 90 mM.

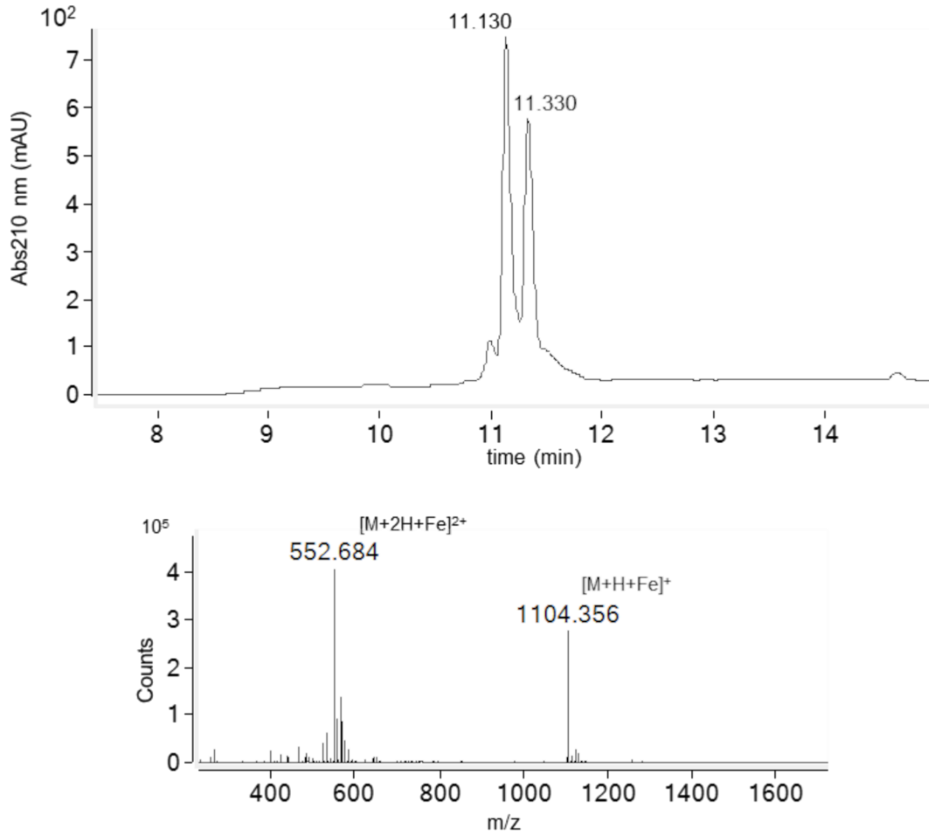

Figure S13. Analysis by LC-MS of pure *cyclo*(-Orn(N^{δ}-CO-C4-AAZTA,γ-mercapto)-Arg-Gly-Asp-D-Phe-). Peptide mass spectrum shows +53 Da ascribable to the chelation of a Fe(III) ion by AAZTA during the LC-MS analysis.

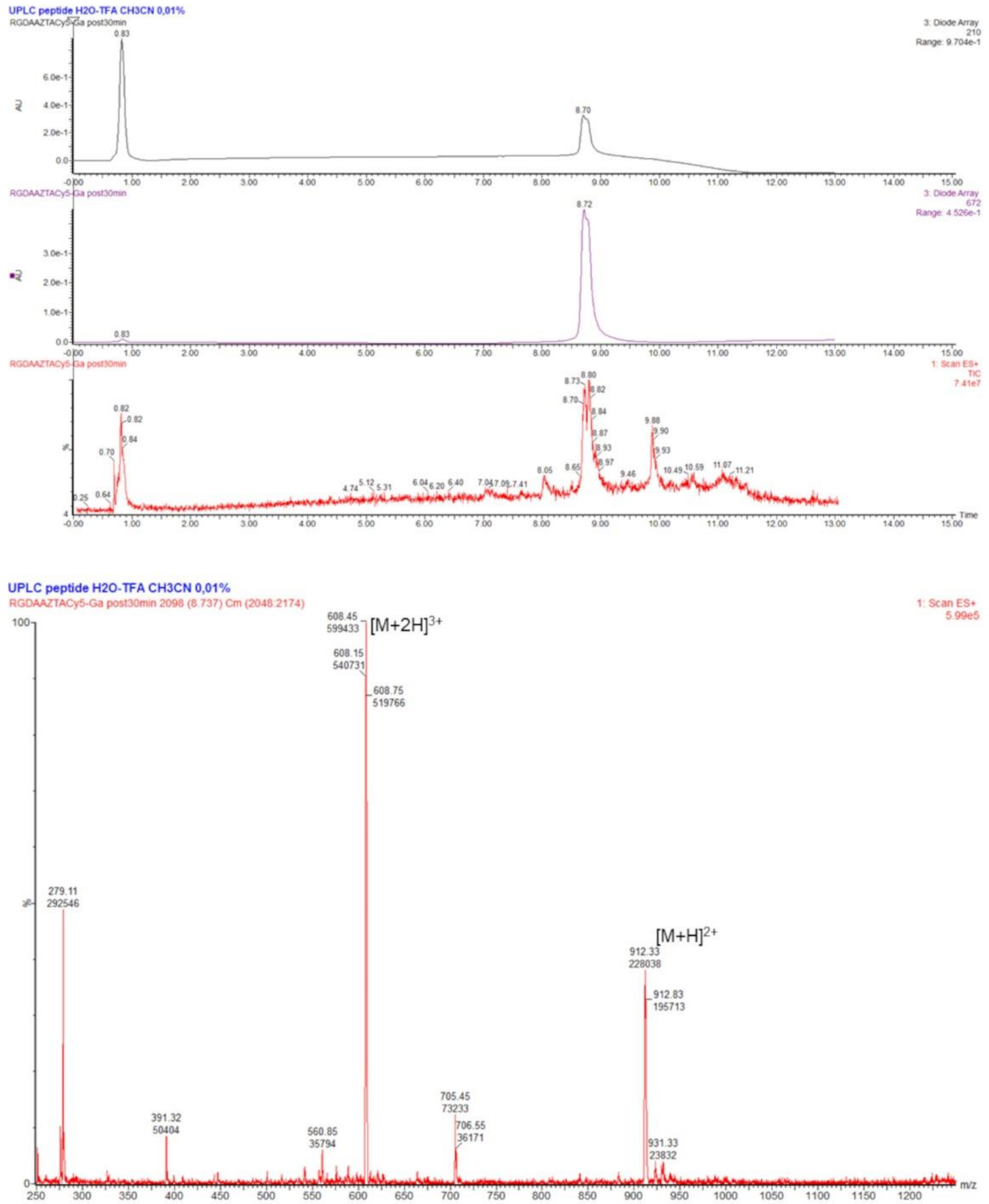

Figure S14. LC-MS analysis of pure *cyclo*(-Orn(N^{δ}-CO-C4-AAZTA,γ-mercapto)-Arg-Gly-Asp-D-Phe-) complexed with Ga(III). Upper panels) chromatographic profiles revealed at 210 nm (black trace), at 672 nm (purple trace) and Total ionic current (TIC) profile (red trace); lower panel) ESI mass spectrum of the peaks eluted at ~ 8,7 min. MW^{th} average: 1.823.75 Da. Cyanine 5.5 harbors a quaternary nitrogen that confers an extra +1 net charge to the ion species.

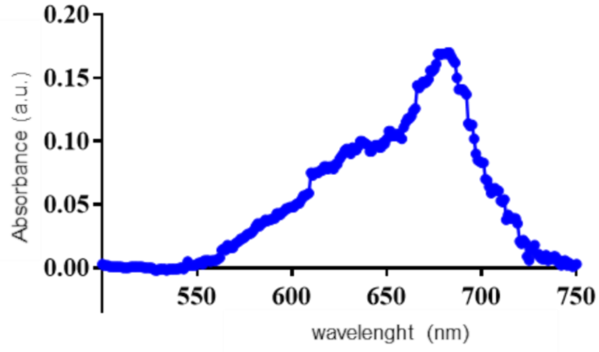

Figure S15. UV-VIS spectrum of *cyclo*(-Orn(N^{δ}-CO-C4-AAZTA,γ-S-succinimido-Cy5.5)-Arg-Gly-Asp-D-Phe-) complexed with Ga(III).
